# Antifibrotic mechanism of avitinib in bleomycin-induced pulmonary fibrosis in mice

**DOI:** 10.1186/s12890-023-02385-9

**Published:** 2023-03-22

**Authors:** Yang Miao, Yanhua Wang, Zhun Bi, Kai Huang, Jingjing Gao, Xiaohe Li, Shimeng Li, Luqing Wei, Honggang Zhou, Cheng Yang

**Affiliations:** 1 State Key Laboratory of Medicinal Chemical Biology, College of Pharmacy and Tianjin Key Laboratory of Molecular Drug Research, Nankai University, Haihe Education Park, 38 Tongyan Road, Tianjin, 300353 People’s Republic of China; 2 Tianjin Key Laboratory of Molecular Drug Research, Tianjin International Joint Academy of Biomedicine, Tianjin, 300457 People’s Republic of China; 3 Tianjin Jikun Technology Co., Ltd. Tianjin, Tianjin, 301700 People’s Republic of China; 4 Tianjin Beichen Hospital, No. 7 Beiyi Road, Beichen District, Tianjin, 300400 People’s Republic of China

**Keywords:** Avitinib, Bleomycin-induced pulmonary fibrosis, TGF-β1/Smad3 signalling, Alveolar epithelial cell injury, Myofibroblast activation

## Abstract

Idiopathic pulmonary fibrosis (IPF) is a chronic, progressive interstitial lung disease characterized by alveolar epithelial cell injury and lung fibroblast overactivation. At present, only two drugs are approved by the FDA for the treatment of IPF, including the synthetic pyridinone drug, pirfenidone, and the tyrosine kinase inhibitor, nintedanib. Avitinib (AVB) is a novel oral and potent third-generation tyrosine kinase inhibitor for treating non-small cell lung cancer (NSCLC). However, the role of avitinib in pulmonary fibrosis has not yet been established. In the present study, we used in vivo and in vitro models to evaluate the role of avitinib in pulmonary fibrosis. In vivo experiments first verified that avitinib significantly alleviated bleomycin-induced pulmonary fibrosis in mice. Further in vitro molecular studies indicated that avitinib inhibited myofibroblast activation, migration and extracellular matrix (ECM) production in NIH-3T3 cells, mainly by inhibiting the TGF-β1/Smad3 signalling pathways. The cellular experiments also indicated that avitinib improved alveolar epithelial cell injury in A549 cells. In conclusion, the present findings demonstrated that avitinib attenuates bleomycin-induced pulmonary fibrosis in mice by inhibiting alveolar epithelial cell injury and myofibroblast activation.

**Supplementary Information:**

The online version contains supplementary material available at 10.1186/s12890-023-02385-9.

## Introduction

Idiopathic pulmonary fibrosis (IPF) is a chronic, progressive and irreversible lung disease characterized by the aberrant accumulation of fibrotic tissue in the lung parenchyma [1, 2]. At present, IPF pathogenesis is not completely understood, but pulmonary fibroblasts and epithelial cells, as the main functional cells, play essential roles in the progression of pulmonary fibrosis [3–5]. Alveoli are important structures of the lung and consist of epithelial cells, and these cells play an essential role in maintaining the size and gas exchange of the lung (oxygen and carbon dioxide) [6–8]. The main role of pulmonary fibroblasts is to participate in lung wound healing, and unlimited fibroblast proliferation can cause excessive repair of the lung, which exacerbates the progression of pulmonary fibrosis and the induction of pulmonary function [8–10].

Transforming growth factor-β1 (TGF-β1) is involved in the development of fibrosis in various organs [11]. TGF-β1 expression levels are increased in IPF patients compared to normal controls, and this cytokine aggravates the progression of pulmonary fibrosis by upregulating Smad and non-Smad signals [11]. Mechanical tension activates the TGF-β signalling pathway in type II alveolar epithelial cells, and reducing this signalling pathway may be a sufficient strategy to alleviate fibrotic progression in IPF patients [12]. Smads from the cell membrane and nucleus have been identified in fibroblasts after TGF-β1 treatment, and Smad3 deficiency attenuates bleomycin-induced pulmonary fibrosis [13]. In addition, TGF-β1 activates non-Smad signalling pathways, such as activating the Akt kinase by targeting PTEN [14], activating the Erk kinase through direct phosphorylation of ShcA [15] and activating the JNK kinase through the FRAF6 protein [16]. TGF-β1 is a potent profibrotic cytokine in foci containing these activated fibroblasts, and it induces fibroblasts to transform into myofibroblasts in the lung [17]. Extracellular matrix (ECM) proteins are an essential determinant of wound healing and are mainly secreted by myofibroblasts, and TGF-β1 significantly promotes fibroblast activation, migration and ECM accumulation in the lungs of IPF patients [18].

Avitinib (AC0010 or AVB) is an irreversible epidermal growth factor receptor (EGFR) inhibitor that selectively targets T790-mutated EGFR, and it is used in the treatment of NSCLC [19]. In addition, avitinib also acts as a novel Bruton’s tyrosine kinase (BTK) inhibitor, which inhibits the phosphorylation of BTK and the PI3K/Akt signalling pathway in mononuclear cell leukaemia (MCL) cells [20]. Previous studies have demonstrated that targeting EGFR and BTK may be an effective method to mediate the development of pulmonary fibrosis [21, 22]. In the present study, we used in vivo and in vitro models to evaluate the role of avitinib in pulmonary fibrosis(Fig. S1), and we further explored the pharmacological mechanism of avitinib in the treatment of lung fibrosis, aiming to provide novel candidate compounds for clinical treatment of lung fibrosis.

## Materials and methods

### Animals

In total, 60 male C57BL/6 J mice (6–8 weeks old and average weight at the start of the experiment was 20–22 g) were purchased from Charles River (Beijing, China). The mice were housed at the Experimental Animal Centre of Nankai University under good growth and living conditions. The care and experimental procedures complied with guidelines approved by the Institutional Animal Care and Use Committee (IACUC) of Nankai University (Permit No. SYXK 2019–0001). All mice were provided unlimited access to food and water, and they were maintained under a 12-h light–dark cycle with 60% ± 2% air humidity at 22 °C–26 °C. C57BL/6 J mice are the predominant animal model for lung injury as this particular strain is highly susceptible to lung injury following intratracheal BLM administration [23, 24].

Bleomycin (Medicine Co., Tokyo, Japan) was dissolved in 0.9% normal saline (Sangon, Shanghai, China) and administered through intratracheal instillation (2 U/kg). Mice in the NaCl group received 0.9% normal saline only by intratracheal instillation. The 60 mice were randomly categorized into the following 6 groups: NaCl group (*n* = 6), bleomycin (2 U/kg) group (*n* = 6), bleomycin + nintedanib (100 mg/kg) group (*n* = 6), bleomycin + avitinib (15 mg/kg) group (*n* = 6), bleomycin + avitinib (30 mg/kg) group (*n* = 6) and bleomycin + avitinib (60 mg/kg) group (*n* = 6). According to other studies and the long-term experimental doses used in our previously published articles [20, 25, 26], we selected the effective and nontoxic concentrations of the drugs for studies of avitinib and nintedanib. The mice were given oral nintedanib (Macklin, Shanghai, China) and avitinib (Gage Bio, Jinan, China), both of which were administered once a day for 7 days (Day 7 to Day 13 after bleomycin administration) [27]. Lung tissues were harvested on Day 14 for subsequent experiments. For anaesthesia, 40 mg/kg pentobarbital sodium was used via intraperitoneal injection [28, 29]. We selected nintedanib as a positive control because nintedanib is one of two drugs approved to treat IPF patients, and it is also used as a positive control agent in many relevant studies.

### Western blot analysis

RIPA buffer (Beyotime, Shanghai, China) containing a protease inhibitor cocktail (Sigma, Rehovot, Israel) was used to lyse cells and tissues, and the protein concentrations of the supernatant were analysed using the BCA Protein Assay kit (Beyotime, Shanghai, China). SDS–PAGE was used to separate proteins based on molecular weight, and all proteins were transferred to polyvinylidene difluoride (PVDF) membranes (Roche Diagnostics, Indianapolis, IN, USA). The membranes were blocked with 5% BSA (Cell Signaling Technology, Beverly, MA, USA) in TBS-T for one hour at room temperature and then incubated overnight at 4 °C with the primary antibodies shown in Table 1. The membranes were then incubated with secondary antibodies for approximately 2 h at room temperature. HRP-labelled goat anti-rabbit IgG (H + L) (Cell Signaling Technology, Beverly, MA, USA) and HRP-labelled goat anti-mouse IgG (H + L) (Cell Signaling Technology, Beverly, MA, USA) were used as the secondary antibodies. Immunoreactivity was detected by ECL (Affinity Biosciences, OH, USA), and the relative density was analysed by ImageJ.

**Table 1:** List of primary antibodies

Antibody name	Item No	Antibody name	Item No
p-Smad2(S467)	Affinity, AF3449	p-Smad3 (S423/425)	Affinity, AF8315
Smad2	Affinity, AF6449	Smad3	Affinity, AF6362
α-SMA	Affinity, BF9212	E-Cadherin	CST, 144,725
Collagen I	Affinity, AF7001	Vimentin	CST, 5741 T
GAPDH	Affinity, AF7021		

### Real-time quantitative PCR

TRIzol (Thermo Scientific Inc., Waltham, MA, USA) was used to extract total RNA from NIH-3T3 and A549 cells and lung tissues. All primers (Table 2) for α-SMA (α-smooth muscle actin), Col1 1 (collagen 1), vimentin and E-cadherin were purchased from Qingke Biological Technology (Beijing, China). RNA transcription was performed using the Reverse SYBR Select Master Mix kit according to the manufacturer’s instructions (Tiangen, Beijing, China). Fluorescence quantitative real-time PCR (Yeasen, Shanghai, China) was subsequently performed.

**Table 2:** List of gene primers

Gene	Forward Primer Sequence (5′-3′)	Reverse Primer Sequence (5′-3′)
α-SMA(mouse)	GCTGGTGATGATGCTCCCA	GCCCATTCCAACCATTACTCC
Col1 a1(mouse)	CCAAGAAGACATCCCTGAAGTCA	TGCACGTCATCGCACACA
E-cadherin(mouse)	CAGCCTTCTTTTCGGAAGACT	GGTAGACAGCTCCCTATGACTG
Vimentin(mouse)	ATGACCGCTTTGCCAACTAC	GTGCCAGAGAAGCATTGTCA
β-actin(mouse)	AGGCCAACCGTGAAAAGATG	AGAGCATAGCCCTCGTAGATGG
E-cadherin(human)	GAGTGCCAACTGGACCATTCAGTA	AGTCACCCACCTCTAAGGCCATC
Vimentin(human)	CCAGGCAAAGCAGGAGTC	GGGTATCAACCAGAGGGAGT
β-actin(human)	GGACTTCGAGCAAGAGATGG	AGCACTGTGTTGGCGTACAG

### Haematoxylin–eosin (H&E) and masson’s trichrome staining and measurement of the pulmonary fibrosis area

Mouse lungs were fixed in 10% formalin for at least 2 days, and different concentrations of dimethylbenzene and ethanol were used to dehydrate lung tissues. Tissues were then embedded in paraffin, and lung Sects. (5 µm) were prepared for haematoxylin–eosin (HE) staining and Masson’s trichrome staining (Solarbio, Shanghai, China). Images of the lung structure were obtained using a fluorescence microscope (Nikon, Japan), and Image-Pro Plus Version 6.0 (Media Cybernetics Inc., Bethesda, MD, USA) was used to calculate the fibrotic area of lung tissues [30]. The entire lung area was demarcated, and the total pixel Pw of the region was automatically calculated. The total pixel Pf of the fibrosis region was then calculated and used to calculate the fibrosis ratio using the following formula: fibrosis ratio = fibrosis area total pixel Pf/total lung total pixel Pw.

### Immunohistochemistry

Immunohistochemical (IHC) analyses of mouse lung sections were performed to evaluate the protein expression levels of α-SMA, Col 1, E-cadherin and vimentin using the UltraSensitive™ SP (Mouse/Rabbit) IHC Kit and DAB Kit (Maxim, Fuzhou, China). In brief, the tissue sections were pretreated with antigen retrieval solution at high temperature (Maxim, Fuzhou, China), blocked, incubated overnight at 4 °C with primary antibodies against the targeted protein, and stained with DAB solution and haematoxylin solution. Images were obtained using a fluorescence microscope (Nikon, Tokyo, Japan).

### Evaluation of pulmonary function

The mice were anaesthetized with 40 mg/kg pentobarbital sodium in 0.9% normal saline solution (i.p.). The mice were fixed to the console, and they were then sprayed with 75% alcohol for disinfection. The fur was cut from the abdomen to the mandible, and the trachea was exposed. The trocar needle was inserted from the cartilage ring and pulled out to keep the trocar tightly fastened. A mouse plethysmography chamber was used to analyse pulmonary function using the AniRes2005 Animal Lung Function Analysis System (Biolab, Beijing, China). The mice were laid in a body box, and a computer was used to assess pulmonary function indices, such as forced vital capacity (FVC), inspiratory resistance (Ri), expiratory resistance (Re), and dynamic compliance (Cydn). The sternum of the mouse was then cut, and the inferior vena cava was cut and ligated from the root of the right lung. The heart was perfused with PBS until the lungs turned white, and the heart was quickly removed. The right lung was removed from the ligation, rinsed with PBS, and placed into an ampoule bottle to determine the hydroxyproline content. A syringe was used to remove the 4% formaldehyde fixative solution from the cannula and put it through the trachea to prop up the left lung until the lung was full. Finally, the trachea and left lung were removed together and fixed in 4% formaldehyde fixative solution, which were used for subsequent lung tissue sectioning and staining.

### Hydroxyproline assay

A frequently used method of analysing hydroxyproline was employed to detect the collagen content of the right lungs of mice. The right lungs were dried and subjected to acid hydrolysis. Concentrated sodium hydroxide was then used to ensure a pH value of 6.5–8.0. Chloramine-T (MERYER, Shanghai, China) spectrophotometric absorbance was employed for hydroxyproline analysis as previously described [31]. Finally, the hydroxyproline level of the mouse right lung was measured using a spectrophotometer at a wavelength of 550 nm.

### Cell culture

Human lung cancer epithelial cells (A549), mouse embryonic fibroblasts (NIH-3T3) and CAGA-NIH-3T3 cells were kindly provided by Professor Wen Ning, School of Life Sciences, Nankai University. NIH-3T3 and CAGA-NIH-3T3 cells were maintained in DMEM (Solarbio, Beijing, China) containing 10% foetal bovine serum (Gibco, Carlsbad, CA, USA) and 1% penicillin–streptomycin (Gibco, Carlsbad, CA, USA). A549 cells were cultured in RPMI 1640 medium (Solarbio, Beijing, China) containing 10% foetal bovine serum and 1% penicillin–streptomycin. NIH-3T3, CAGA-NIH3T3 and A549 cells in DMEM or RPMI 1640 medium with 0.1% FBS were stimulated with TGF-β1 (human TGF-beta1-mammalian; Lianke Biotechnology, Hangzhou, China) or avitinib (dissolved in DMSO). All cells were cultured in an incubator (PHCBI, Tokyo, Japan) with 5% CO_2_ at 37 °C.

### Wound-healing assays

NIH-3T3 cells were seeded into 6-well plates for the wound-healing assay, and 200-µL sterile pipette tips were used to generate a wound. Cells were cultured in AVB (1.25 and 2.5 µM) and/or TGF-β1 (5 ng/mL) in DMEM with 0.1% FBS. Images were obtained at 0, 12, and 24 h by using a light microscope (Nikon, Tokyo, Japan).

### Cell viability analysis

NIH-3T3 cells were seeded into a 96-well plate and allowed to adhere. The cell culture medium was then replaced with complete medium containing avitinib at a series of concentrations (0.16 μM- 2.5 μM), and the cells were incubated at 37 °C for 24 h. On the second day, 20 μL of thiazole blue tetrazole bromide (MTT, 5 mg/mL, Keygene Biotechnology, Nanjing, China) was added to each well followed by incubation at 37 °C for 4 h. Finally, 150 μL of DMSO was added to each well at room temperature, and the absorbance was read at 570 nm by a microplate reader. Cell viability (%) was used to evaluate the ratio of viable cells.

### Luciferase assay

In the present study, a luciferase luminescence reporting system was established for the TGF-β1/Smad3 pathway. CAGA-NIH-3T3 cells were established by stably transfecting a plasmid containing 12 copies of the Smad3-binding sequence (CAGA) in front of the luciferase reporter gene upstream of the TGF-β1 promoter; when the CAGA-NIH 3T3 cells were stimulated by TGF-β1, phosphorylated Smad3 was nucleated and bound to CAGA, and luciferase was transcribed and translated, thus resulting in an increase in the luminescence measurement value. Cells were seeded in 96-well plates and cultured overnight. After serum starvation for approximately 20 h, cells were treated with TGF-β1 (5 ng/mL) and/or avitinib (0, 0.16, 0.31, 0.62, 1.25, and 2.5 μM) in DMEM with 0.1% FBS for 18 h. The lysates were used to determine luciferase activity with a luminescence detection system (Promega, Madison, WI, USA) according to the manufacturer’s instructions. Total light emission of plates was detected using a GloMax®-Multi Detection System (Promega, Madison, USA).

### Immunofluorescence

NIH-3T3 cells were treated with TGF-β1 (5 ng/mL) and/or avitinib (1.25 and 2.5 µM) in DMEM with 0.1% FBS for 24 h. Cells were then fixed with 4% paraformaldehyde for 15 min, permeabilized with 0.2% Triton X-100, blocked with 5% BSA, and then incubated with α-SMA primary antibody overnight at 4 °C. Cells were then washed three times with 1 × PBS-T (3 min per wash) and incubated with fluorescein (FITC)-conjugated AffiniPure goat anti-mouse IgG (H + L) (Jackson Immunoresearch, West Grove, PA, USA) for 2 h at room temperature in the dark. Cellular nuclei were stained with DAPI solution (Solarbio, Beijing, China), and cells were imaged using a Leica TCS SP8 confocal laser scanning microscope (Leica, Germany).

### Statistical analysis

The results were analysed by Prism software (version 7.0) and are presented as the mean ± SD. One-way ANOVA was used to analyse significant differences and concordance. The variance homogeneity test was conducted on the data before one-way ANOVA. Statistical analysis was performed with subsequent Bonferroni correction, and comparisons between different groups were performed. p < 0.05 was considered statistically significant.

## Results

### Avitinib reduces bleomycin-induced pulmonary fibrosis in C57BL/6 J mice

**Fig. 1:**
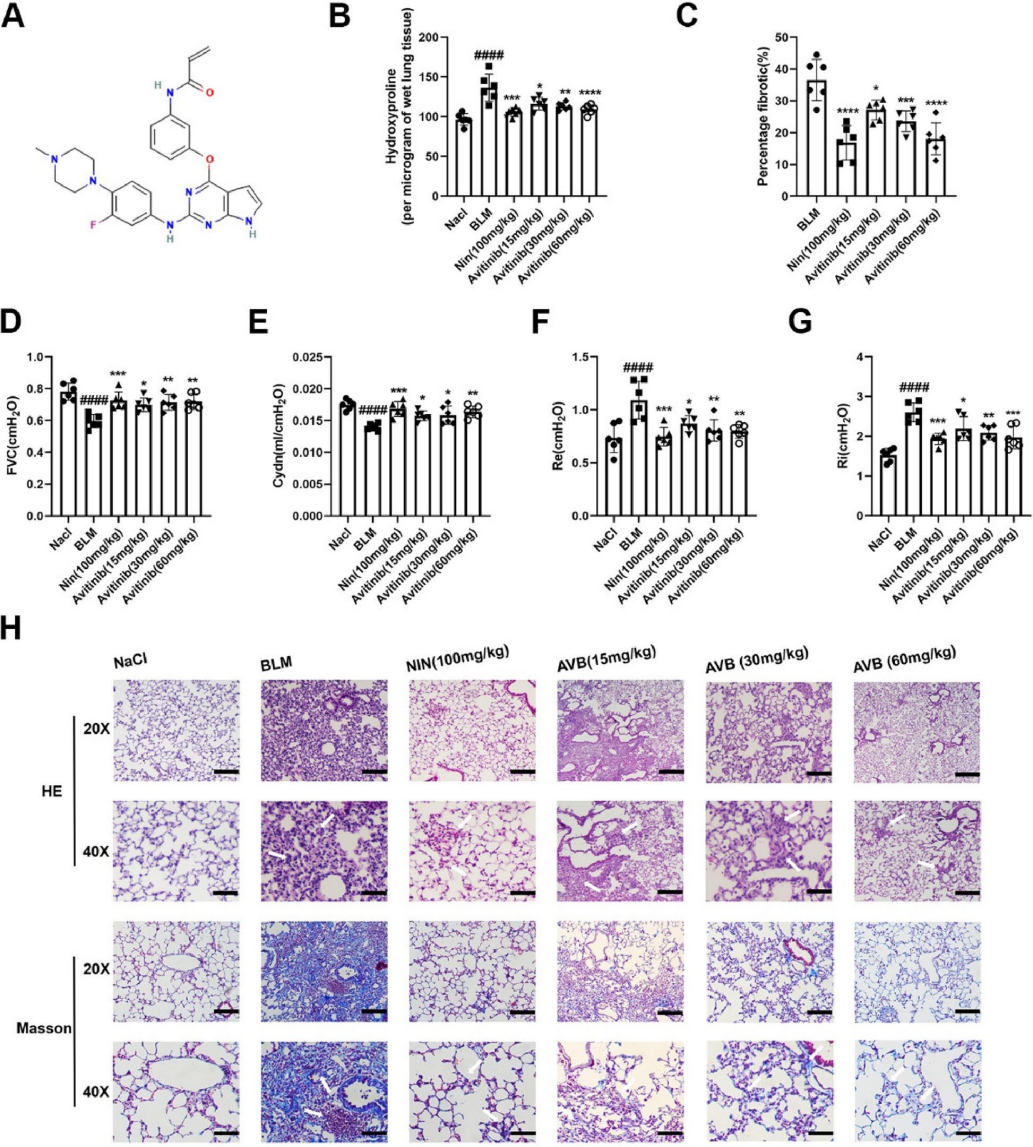
Avitinib reduced bleomycin-induced pulmonary fibrosis in C57BL/6 J mice. The mice were intratracheally treated bleomycin (2U/kg) on day 0, AVB (15, 30 and 60 mg/kg) and Nin (100 mg/kg) were administrated orally from days 7– 13, lungs of mice were harvested on day 14. **A** Structure of Avitinib (The structural formula is quoted from Pubchem). **B** Hydroxyproline contents in right lung tissues. **C** Quantitative analysis of fibrotic area in left lung tissues. **D-G** Pulmonary function tests, including forced vital capacity (FVC), dynamic compliance (Cydn), expiratory resistance (Re) and inspiratory resistance (Ri). **H** Hematoxylin–eosin (H&E) staining of left lung tissue sections for tissue structures and Masson staining was used to analyze the collagen contents in left lung sections. Scale bars:50 µM. Data was noted as the means ± SD, *n* = 6. ^*####*^*P* < 0.0001 versus NaCl group. *P < 0.05, **P < 0.01, ***P < 0.001, ****P < 0.0001 versus BLM group

To evaluate whether avitinib has a potential effect on bleomycin-induced pulmonary fibrosis, we established an animal disease model of pulmonary fibrosis by intratracheally treating mice with bleomycin. The structure of avitinib was shown in Fig. 1A. The results demonstrated that avitinib significantly reduced the hydroxyproline content of the right lungs and the percentage of fibrotic tissues in mice (Fig. 1B-C). Pulmonary function is a key evaluation index used in the treatment of IPF patients, and avitinib improved pulmonary function parameters, including FVC, Cydn, Re and Ri. FVC and Cdyn were increased in avitinib-treated mice compared to the model group, whereas Ri and Re were decreased in avitinib-treated mice compared to the model group (Fig. 1D-G). HE and Masson’s trichrome staining of the lung sections showed that avetinib improved alveolar structure and reduced collagen production (Fig. 1H). In addition, avitinib alleviated the progression of pulmonary fibrosis in a dose-dependent manner, and its effect at high doses (60 mg/kg) was similar to that of the positive control drug, nintedanib. Therefore, these findings indicated that avitinib alleviates pulmonary fibrosis in a bleomycin-induced mouse model by improving pulmonary function and reducing the percentage of fibrotic tissue and hydroxyproline content.

### Avitinib slows TGF-β1-induced migration of NIH-3T3 cells in vitro

**Fig. 2:**
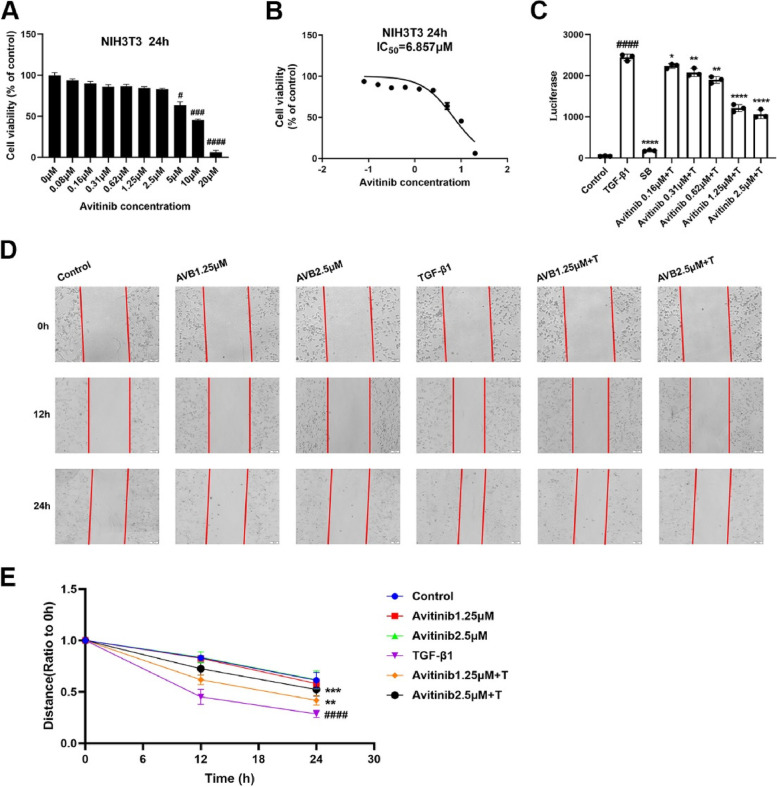
Avitinib slows TGF-β1-induced migration of NIH-3T3 cells in vitro. **A** MTT assays of NIH-3T3 cells. Cells were exposed to the indicated doses of AVB (0.08 to 20 µM) for 24 h. **B** IC50 = 6.857 µM. **C** CAGA-mouse embryonic fibroblast NIH-3T3 cells were treated with TGF-β1 (5 ng/mL) and a series concentration (0.16, 0.31, 0.63, 1.25, 2.5 µM) of AVB for 18 h. Quantitative analyses are shown beside.**D** TGF- β1 (5 ng/mL) and/or AVB (1.25, 2.5 µM) incubated with NIH-3T3 cells for 0 h, 12 h, 24 h. Scale bars: 100 μm.(E) Distance analyses are shown below. Data was noted as the means ± SD, *n* = 3. ^*#*^*P* < 0.05, ^*###*^*P* < 0.001, ^*####*^*P* < 0.0001 versus Control group. *P < 0.05, **P < 0.01, ****P < 0.0001 versus TGF-β1 group

The migration of lung fibroblasts plays an important role in the pathological process of pulmonary fibrosis. NIH-3T3 cells were exposed to medium with or without TGF-β1 and various doses of avetinib for 24 h. The results showed that when the concentration of avitinib was less than 2.5 μM, there was no obvious toxic effect in normal (NIH-3T3) cells (Fig. 2A), and the IC50 value of avitinib was 6.857 μM (Fig. 2B). When the concentration of avitinib was less than 2.5 μM, the number of living cells did not significantly decrease, and there was no significant difference compared to the control group, which indicated that avitinib had no effect on cell viability. Thus, a concentration of 2.5 μM was considered to belong to the safe concentration range. As shown in Fig. 2C, avitinib inhibited the TGF-β1/Smad3 signalling pathway in a dose-dependent manner. Combined with the above results, we selected two concentrations (1.25 μM and 2.5 μM) for subsequent experiments. In the wound-healing experiments, avitinib inhibited TGF-β1-induced migration of NIH-3T3 cells in a dose-dependent manner (Fig. 2D-E).

### Avitinib inhibits fibroblast activation via the TGF-β1/Smad signalling pathway in NIH-3T3 cells

**Fig. 3:**
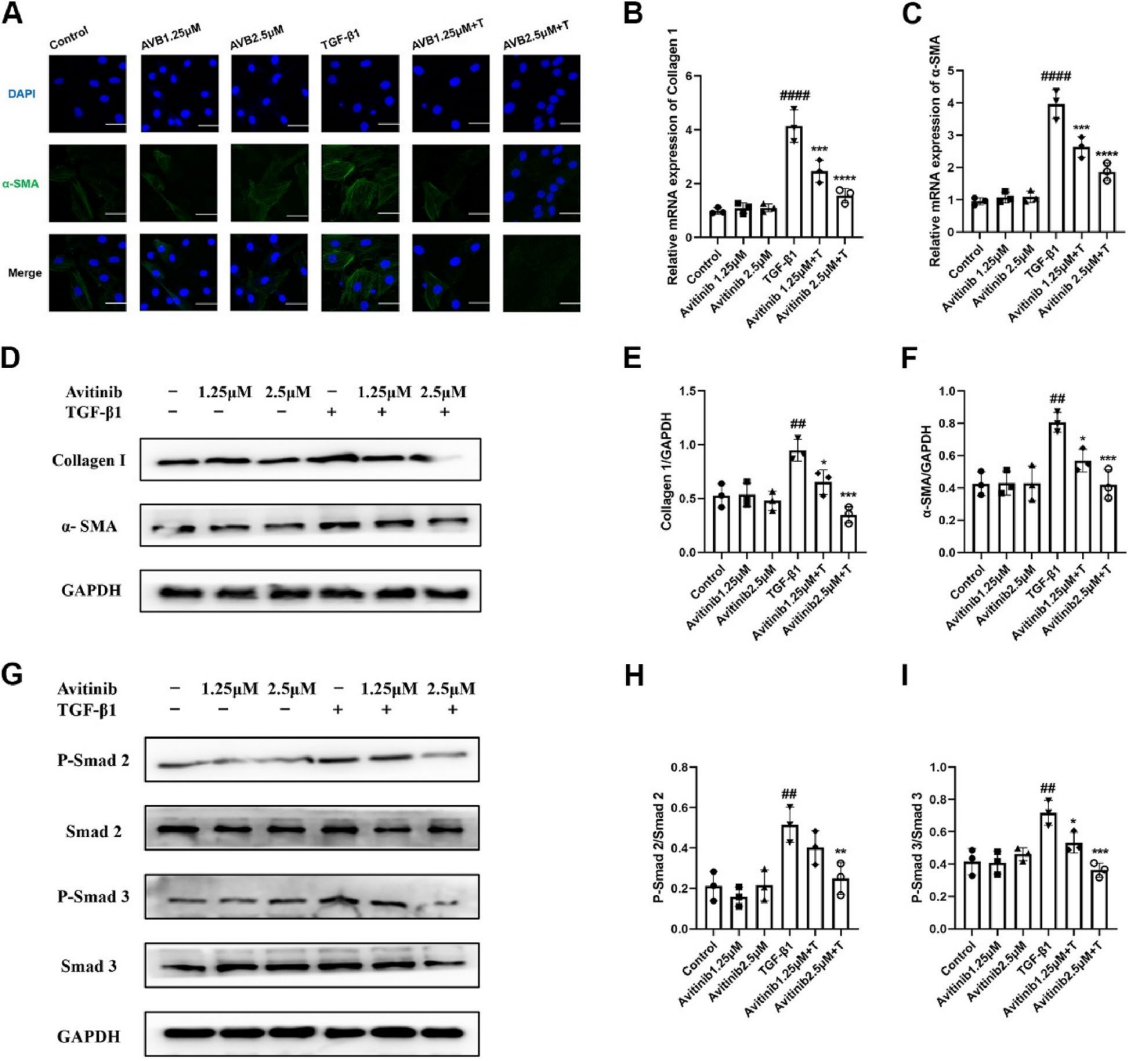
Avitinib inhibits fibroblasts activation via TGF-β1/Smad signaling pathway in NIH-3T3 cells. AVB (1.25, 2.5 µM) and/or TGF-β1 (5 ng/mL) was exposed to NIH-3T3 cells for 24 h, and the α-SMA expression level was detected by cellular immunofluorescence. Scale bar:50 µm;(**B-C**) AVB (1.25, 2.5 µM) and/or TGF- β1 (5 ng/mL) was exposed to NIH-3T3 cells for 12 h, and quantitative real-time PCR was used to analyze the mRNA transcription level of Col 1 and α-SMA. **D-F** TGF-β1 (5 ng/mL) and/or AVB (1.25,2.5 µM) were exposed to NIH-3T3 cells for 24 h, and the Col 1 and α-SMA expression levels were detected by Western Blot. Quantitative analyses of Western blot are shown beside. **G-I** NIH-3T3 cells were exposed to TGF- β1 (5 ng/mL) for 0.5 h and AVB (1.25, 2.5 µM) for 2 h, and detecting the protein expression levels of Smad3, Smad2, p-Smad2 (S467) and p-Smad3 (S423/425) by Western blot. Densitometric analyses are shown beside. Data was presented as the means ± SD, *n* = 3. ^*##*^*P* < 0.01, ^***####***^*P* < 0.0001 versus control group. *P < 0.05, **P < 0.01, ***P < 0.001, ****P < 0.0001 versus TGF-β1 group

As shown in Fig. 3A, TGF-β1 induced the expression of α-SMA in NIH-3T3 cells as indicated by a significant increase in the fluorescence intensity of the protein. Avitinib inhibited the expression of α-SMA, but avitinib had no obvious effect on normal 3T3 cells.

Similarly, avitinib downregulated TGF-β1-induced mRNA transcription of Col 1 and α-SMA in NIH-3T3 cells (Fig. 3B-C). In addition, TGF-β1 promoted Col 1 and α-SMA expression, whereas avitinib inhibited the expression of these proteins in NIH-3T3 cells (Fig. 3D-F), which indicated that avitinib inhibited TGF-β1-induced fibroblast activation in NIH-3T3 cells. In addition, avitinib significantly downregulated the levels of Smad3 (S423/425) and Smad2 (S467) phosphorylation in NIH-3T3 cells (Fig. 3G-I). Therefore, these findings demonstrated that avitinib inhibits TGF-β1-induced fibroblast activation and ECM production in a dose-dependent manner, mainly by downregulating the TGF-β1/Smad signalling pathway.

### Avitinib inhibits bleomycin-induced fibroblast activation and extracellular matrix (ECM) production in vivo

**Fig. 4:**
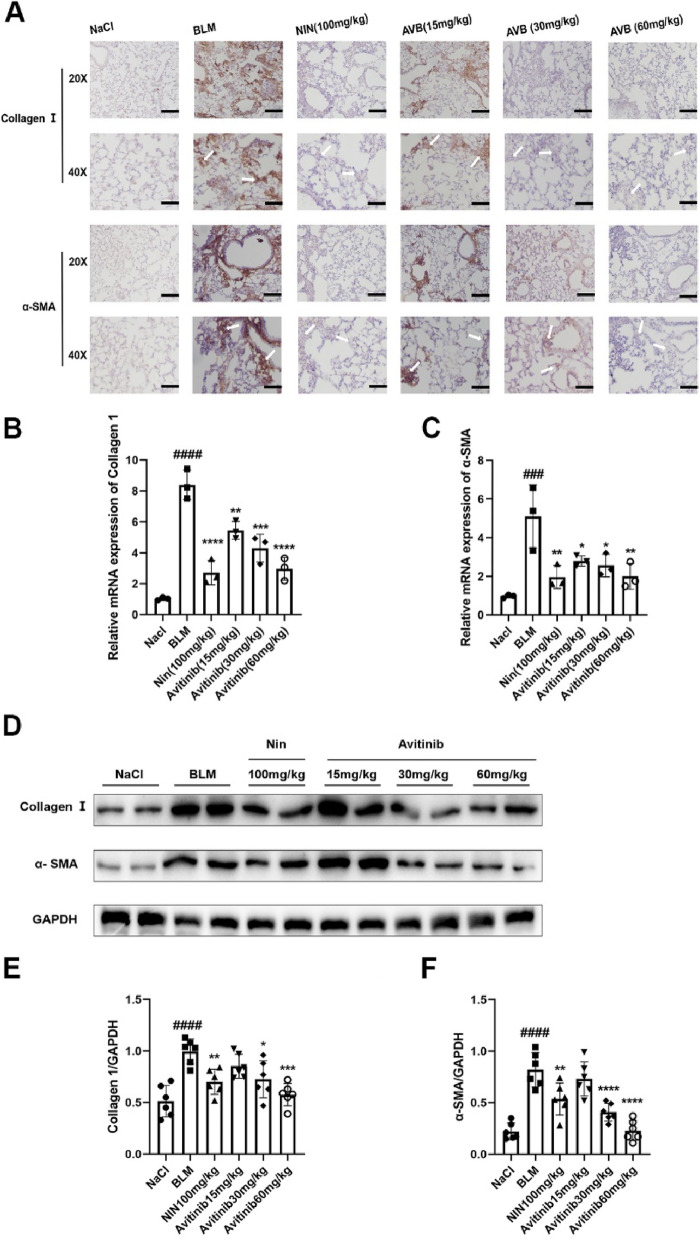
Avitinib inhibits bleomycin-induced fibroblasts activation and Extracellular matrix (ECM) production in vivo. BLM (2U/kg) was intratracheally given at day 0, and Nin (100 mg/kg) and AVB (15, 30 and 60 mk/kg) were orally treated at day 7–13 and harvested at day 14. **A** The expression levels of Col 1 and α-SMA were analyzed by immunohistochemistry in left lung sections. Scale bars: 50 μM. **B-C** The mRNA translation levels of Col 1 and α-SMA in lung tissue homogenates. **D-F** Western Blot was used to analyze the protein expression levels of Col 1 and α-SMA in lung tissue homogenates. Densitometric analyses are shown beside. Data was noted as the means ± SD, *n* = 6. ^*###*^*P* < 0.001, ^*####*^*P* < 0.0001 versus NaCl group. *P < 0.05, **P < 0.01, ***P < 0.001, ****P < 0.0001 versus BLM group

For the in vivo experiments, immunohistochemistry analysis indicated that avitinib decreased collagen I and α-SMA expression levels in lung sections (Fig. 4A). At the same time, avitinib inhibited Col 1 and α-SMA mRNA translation levels and protein expression levels in lung tissue homogenates (Fig. 4B-F). In addition, the effect of high-dose avitinib was similar to that of the positive control drug, nintedanib. Thus, the in vivo results showed that avitinib suppresses fibroblast activation and ECM accumulation.

### Avitinib improves alveolar epithelial cell injury in A549 cells

**Fig. 5:**
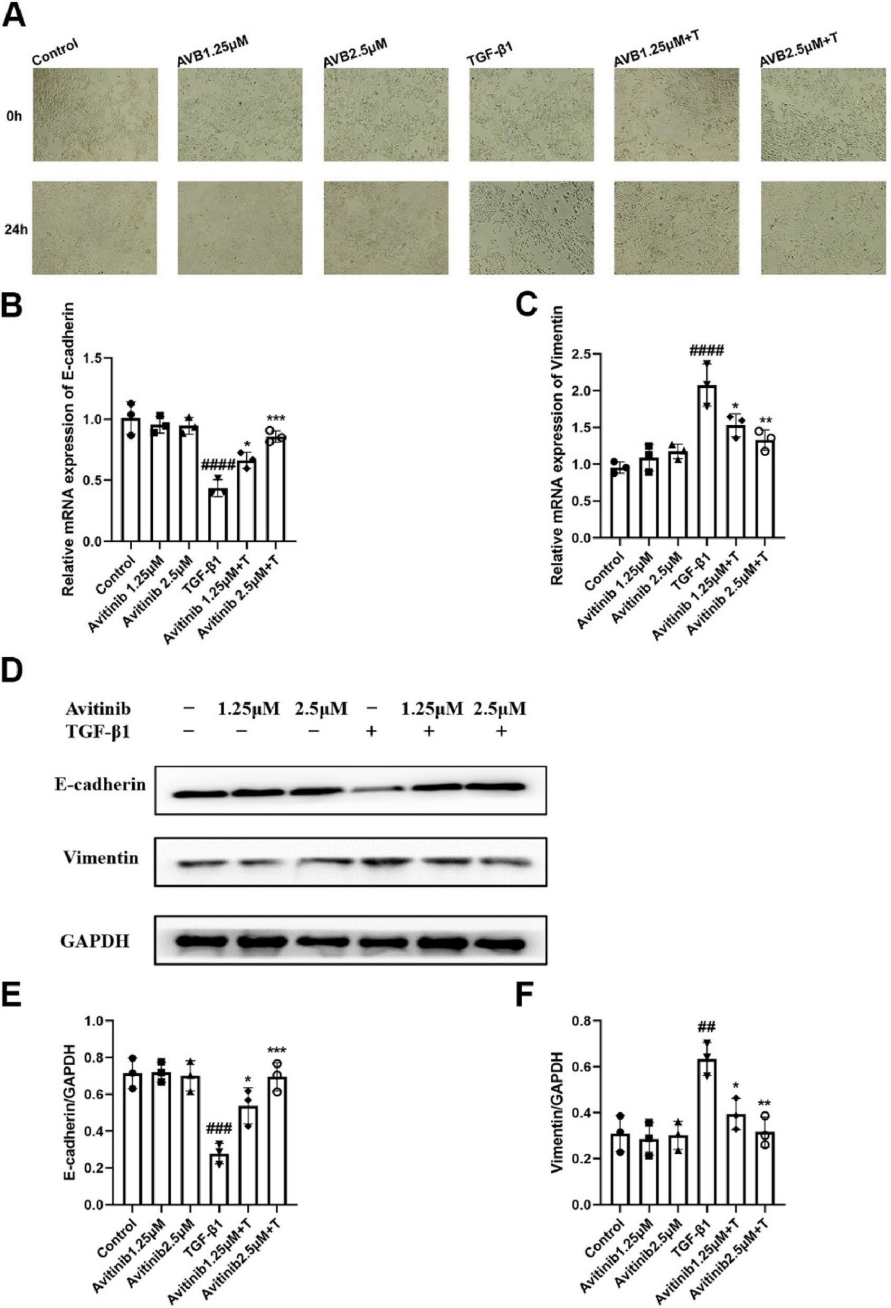
Avitinib improves alveolar epithelial cell injury in A549 cells. **A** AVB significantly inhibited TGF-β1-induced morphological changes in A549 cells. Scale bars: 100 μM. **B-C** AVB (1.25, 2.5 µM) and/or TGF- β1 (5 ng/mL) was exposed to A549 cells for 24 h, and quantitative real-time PCR was used to analyze the mRNA transcription level of E-cadherin and Vimentin. **D-F** TGF-β1 (5 ng/mL) and/or AVB (1.25,2.5 µM) were exposed to A549 cells for 25 h, and the E-cadherin and Vimentin expression levels were detected by Western Blot. Quantitative analyses of Western blot are shown beside. Data was presented as the means ± SD, *n* = 3. ^*##*^*P* < 0.01, ^***###***^*P* < 0.001, ^*####*^*P* < 0.0001 versus control group. *P < 0.05, **P < 0.01, ***P < 0.001 versus TGF-β1 group

Treatment with TGF-β1 for 24 h inhibited A549 cell morphological changes, and avitinib significantly improved this condition in vitro (Fig. 5A). In addition, different concentrations of avitinib did not significantly affect the morphology of normal A549 cells. Avitinib increased E-cadherin mRNA levels but decreased vimentin mRNA levels (Fig. 5B-C), and both E-cadherin and vimentin protein expression levels exhibited the same results (Fig. 5D-F). Hence, these results demonstrated that avitinib significantly improves alveolar epithelial cell injury by regulating E-cadherin and vimentin expression.

### Avitinib improves bleomycin-induced alveolar epithelial cell injury in vivo

**Fig. 6:**
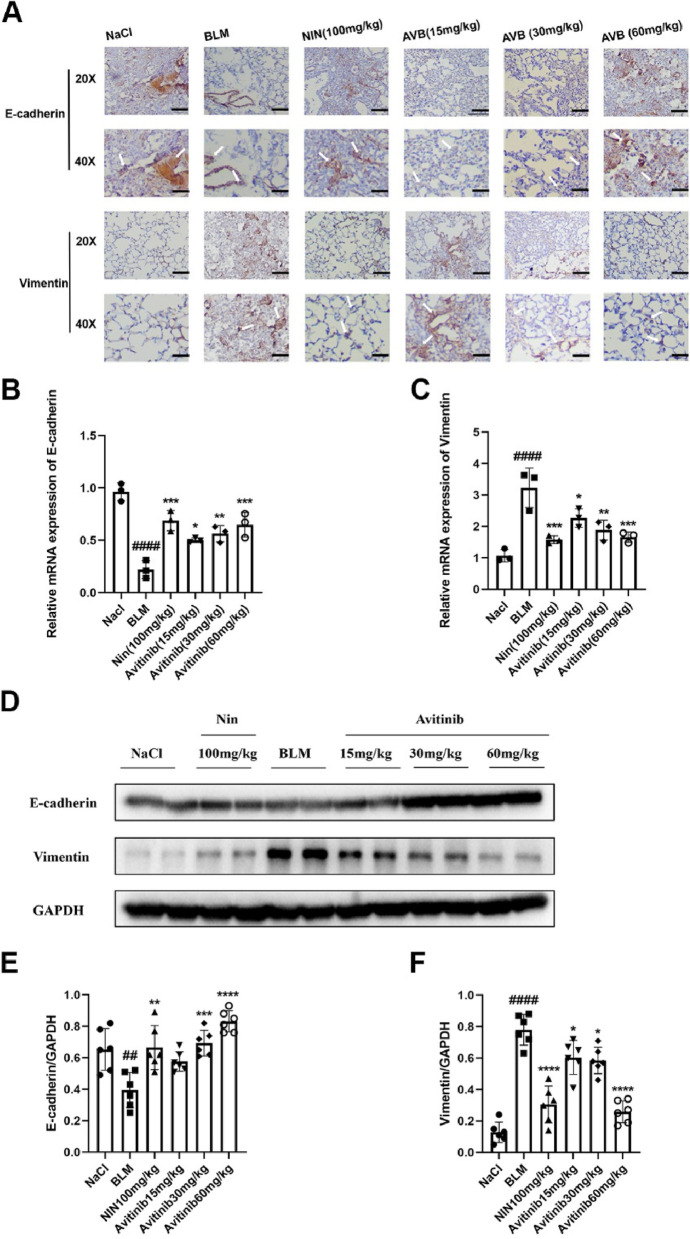
Avitinib improves bleomycin-induced alveolar epithelial cell injury in vivo. BLM (2U/kg) was intratracheally given at day 0, and Nin (100 mg/kg) and AVB (15, 30 and 60 mk/kg) were orally treated at day 7–13 and harvested at day 14. **A** The expression levels of E-cadherin and Vimentin were analyzed by immunohistochemistry. Scale bars: 50 μm. **B-C** The mRNA translation levels of E-cadherin and Vimentin in lung tissue homogenates. **D-F** Western Blot was used to analyze the protein expression levels of E-cadherin and Vimentin in lung tissue homogenates. Densitometric analyses are shown beside. Data was noted as the means ± SD, *n* = 6. ^*##*^*P* < 0.01, ^*####*^*P* < 0.0001 versus NaCl group. *P < 0.05, **P < 0.01, ***P < 0.001, ****P < 0.0001 versus BLM group

The immunohistochemistry results showed that avitinib promoted the expression of E-cadherin and inhibited the expression of vimentin in lung tissue sections in vivo (Fig. 6A). Subsequently, avitinib promoted E-cadherin mRNA transcription and inhibited vimentin mRNA transcription in lung tissue homogenates (Fig. 6B-C). Western blot analysis yielded the same results (Fig. 6D-F). In addition, the effect of high-dose avitinib was similar to that of the positive control drug, nintedanib. Collectively, these data demonstrated that avitinib improves alveolar epithelial cell injury in a pulmonary fibrosis model in vivo.

## Discussion

Idiopathic pulmonary fibrosis is a complex lung disease with an unknown pathogeny, and the administration of bleomycin in a mouse model was used to evaluate the effect of antifibrotic strategies and assess pulmonary fibrogenesis [28]. In an animal model of bleomycin-induced pulmonary fibrosis, avitinib significantly improved pulmonary function and alveolar collapse, and it decreased the content of hydroxyproline, which is a main component of collagen. The IPF lung is characterized by collagen deposition, and reversing this condition alleviates fibrotic development [32]. FVC is a pulmonary functional parameter that reflects parenchymal abnormalities in IPF patients, and FVC changes indicate disease progression [33, 34]. Nintedanib has been approved for the treatment of IPF by the Food and Drug Administration (FDA), and this drug significantly reduces the annual rate of FVC decline compared to placebo in clinical tests [35]. In the present study, nintedanib served as a positive control drug in the in vivo experiments, and the results showed that the anti-pulmonary fibrosis effect of high concentrations of avitinib was equivalent to that of nintedanib, indicating that avitinib has potential to be developed as a therapeutic drug for bleomycin-induced pulmonary fibrosis. Because the efficacy of the drug at the animal level was different from the therapeutic effect at the clinical level, we will continue to compare and evaluate the anti-pulmonary fibrosis efficacy of avitinib based on dosage adjustments and drug safety profiles.

TGF-β1 is a core regulator in the fibrotic progression of IPF patients [36], and canonical TGF-β1 signalling regulates the expression of fibrotic factors via phosphorylation of Smad transcription factors [37]. Smad3 plays an essential role in TGF-β1 signalling. Specifically, Smad3 interacts with Smad4, the shared R-Smad partner, to regulate fibrosis-related transcription factors [11, 38, 39]. As a receptor-regulated Smad (R-Smad), Smad3 often functions with Smad2 and interacts with Smad4 in cells. In view of the role of the two R-Smads, changes in their protein levels are often studied. Pulmonary fibroblasts are involved in wound healing after lung injury, and fibroblasts from IPF patients exhibit the characteristics of stress activation and migration compared to those obtained from normal controls [11]. ECM proteins are mainly secreted by pulmonary fibroblasts, and their overexpression can lead to irreversible scar formation in IPF lungs. Pulmonary fibroblast activation and migration as well as ECM accumulation are mainly regulated by the TGF-β1/Smad3 signalling pathway [40, 41]. In addition, some small molecule compounds (nintedanib, pirfenidone, regorafenib, anlotinib, and imatinib) have been shown to alleviate bleomycin-induced pulmonary fibrosis by downregulating the TGF-β1/Smad3 signalling pathway [26, 34, 42–44]. Therefore, these results indicated that avitinib significantly attenuates bleomycin-induced pulmonary fibrosis mainly by suppressing TGF-β1/Smad signalling in vivo, but further in vitro mechanistic studies are needed.

In cellular and animal experiment results, avitinib positively regulates E-cadherin expression and negatively regulates vimentin expression after TGF-β1 or bleomycin simulation, thereby significantly improving epithelial cell injury. Epithelial cell dysfunction plays an essential role in fibrotic progression [45]. Chronic inflammatory conditions in the lung tissue contribute to epithelial cell injury, and injured cells secrete various profibrotic factors that promote mesenchymal cell migration, proliferation and activation in the fibrotic foci of the lung [46]. Vimentin protein promotes the invasiveness of IPF fibroblasts, and TGF-β1 upregulates vimentin expression [47, 48]. E-cadherin is a transmembrane glycoprotein that mediates cell‒cell adhesion in alveolar epithelial cells, and TGF-β1 decreases E-cadherin expression levels [49, 50]. At the same time, previous studies have found that changes in E-cadherin and vimentin protein expression in human and mouse lung epithelial cells promote the EMT process of fibrosis [51]. Therefore, these findings suggest that avitinib also participates in the related EMT process through E-cadherin and vimentin.

In conclusion, avitinib alleviates bleomycin-induced pulmonary fibrosis and inhibits lung fibroblast activation, migration and ECM accumulation by inhibiting TGF-β1/Smad3 signalling. In addition, avitinib improves epithelial cell injury by regulating vimentin and E-cadherin expression. The antifibrotic pharmacological activity of avitinib suggests that it has a variety of roles other than antitumour effects and may represent a candidate drug for the treatment of pulmonary fibrosis. However, the present study had limitations. For instance, we did not investigate the detailed mechanism of the effect of avitinib on the TGF-β-mediated Smad3 signalling pathway, and the direct target of avitinib in the anti-pulmonary fibrosis process remains unclear. Therefore, the precise effect of avitinib on the TGF-β signalling pathway should be investigated in the future. Moreover, further studies are needed to determine whether avitinib affects other signalling pathways related to pulmonary fibrosis, such as the recognized targets of EGFR and BTK.

## Supplementary Information

**Additional file 1.** AVB original data.

**Additional file 2:** **Fig S1. **In vivo and in vitro experimental schemes in this study.

**Additional file 3.** Western Blot original gels.

## Data Availability

All data in this study are provided by Professor Honggang Zhou, with contact information: E-mail: honggang.zhou@nankai.edu.cn. Add: No.38 Tongyan Road, Jinnan district, Tianjin(300,350), China. Tel: + 86–22-85,358,566. All data are original data of the laboratory, all materials are owned by the laboratory. The datasets used and/or analysed during the current study available from the corresponding author on reasonable request.
