# Supplementary figures and images for: Antifibrotic mechanism of avitinib in bleomycin-induced pulmonary fibrosis in mice
Source: BMC Pulm Med. 2023 Mar 22;23:94. doi: 10.1186/s12890-023-02385-9 (PMC10031887; doi:10.1186/s12890-023-02385-9)

**Supplementary data**


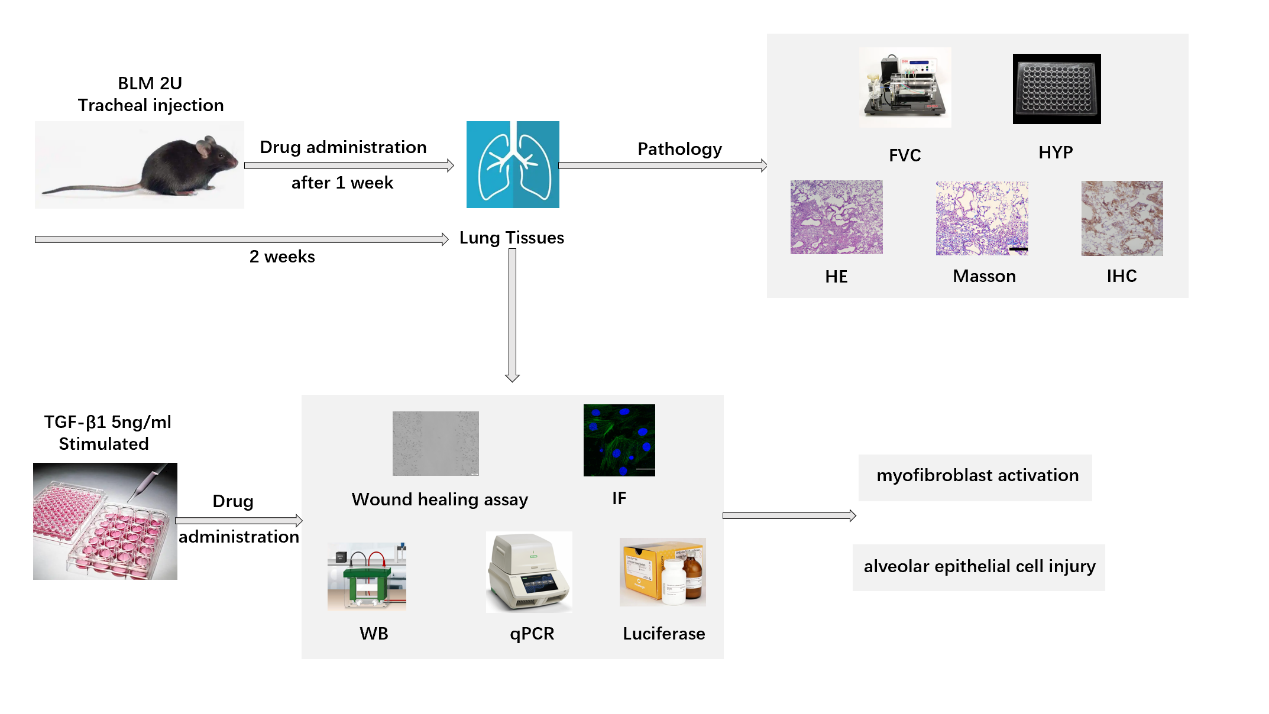


**Fig S1.*****In vivo* and *in vitro* experimental schemes in this study**

Supplement: Supplementary file 2 — Additional file 2: Fig S1. In vivo and in vitro experimental schemes in this study. [file 12890_2023_2385_MOESM2_ESM.docx]
