# Supplementary material for: Antifibrotic mechanism of avitinib in bleomycin-induced pulmonary fibrosis in mice
Source: BMC Pulm Med. 2023 Mar 22;23:94. doi: 10.1186/s12890-023-02385-9 (PMC10031887; doi:10.1186/s12890-023-02385-9)
Supplement: Supplementary file 3 — Additional file 3. Western Blot original gels. [file 12890_2023_2385_MOESM3_ESM.docx]

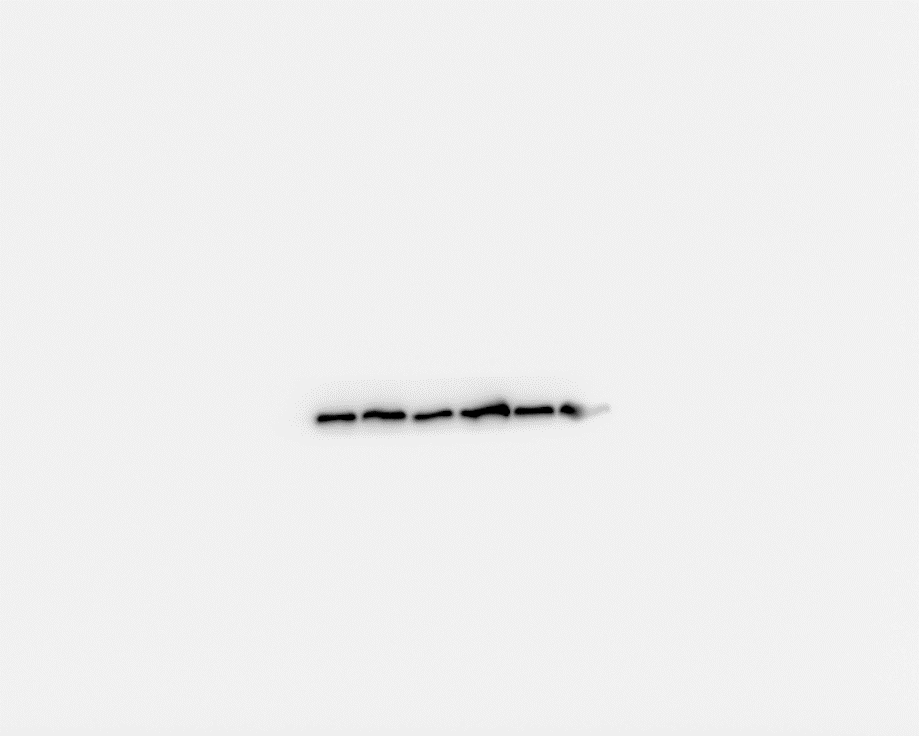


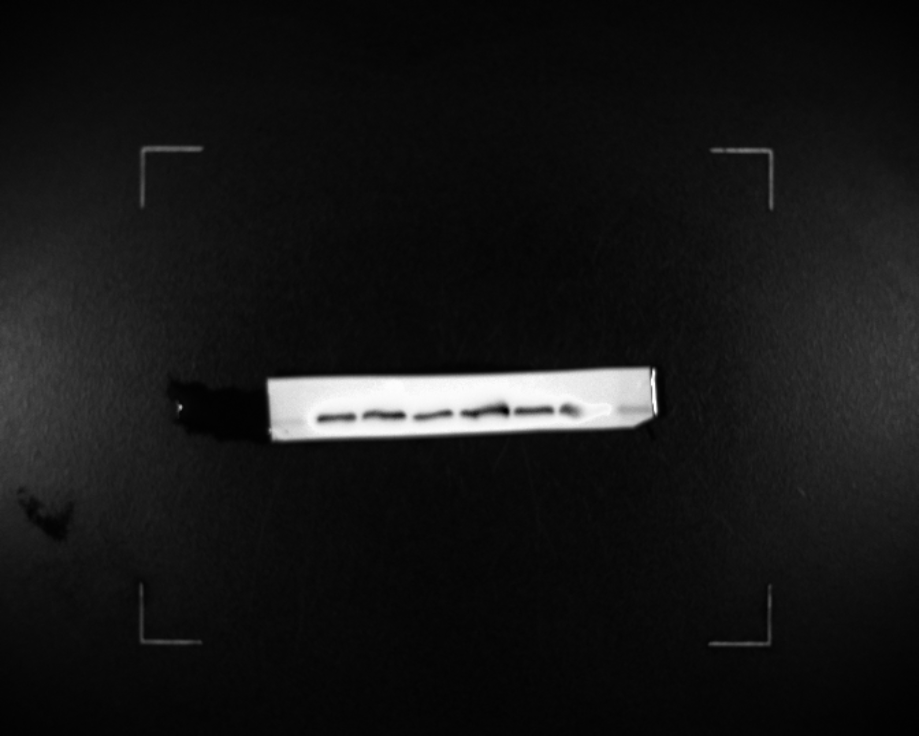


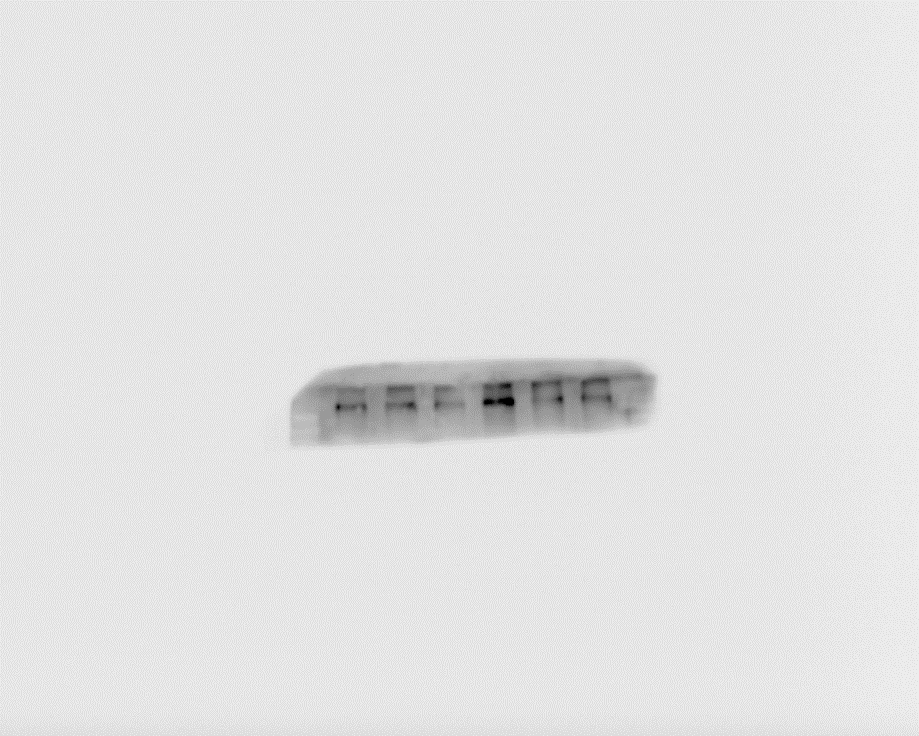


**Figure 3**. The entire original gel of Col 1-manuscript in Figure 3D.


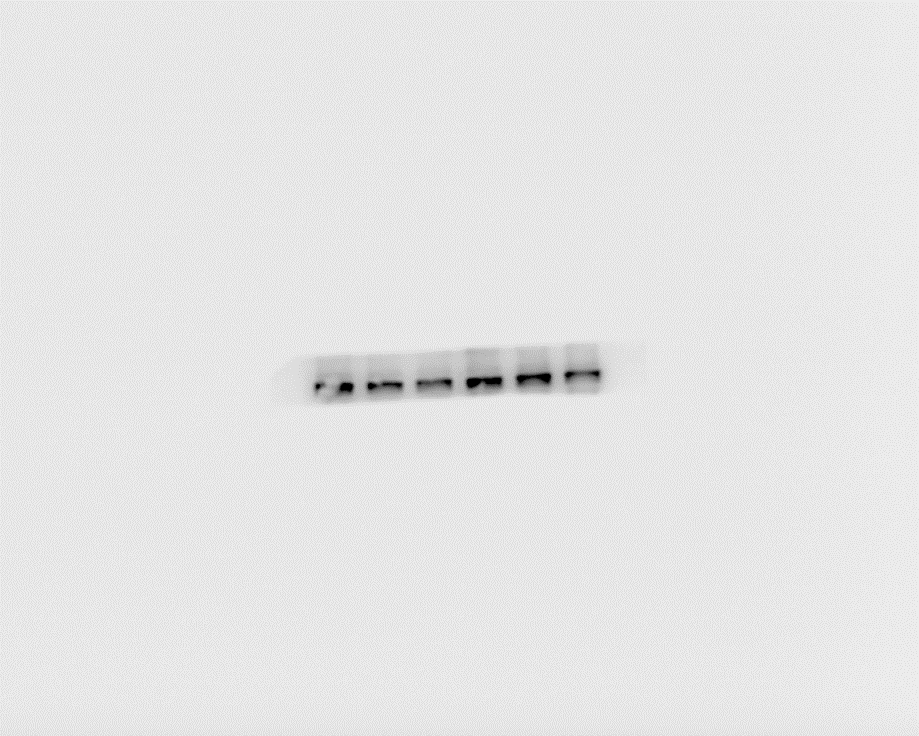


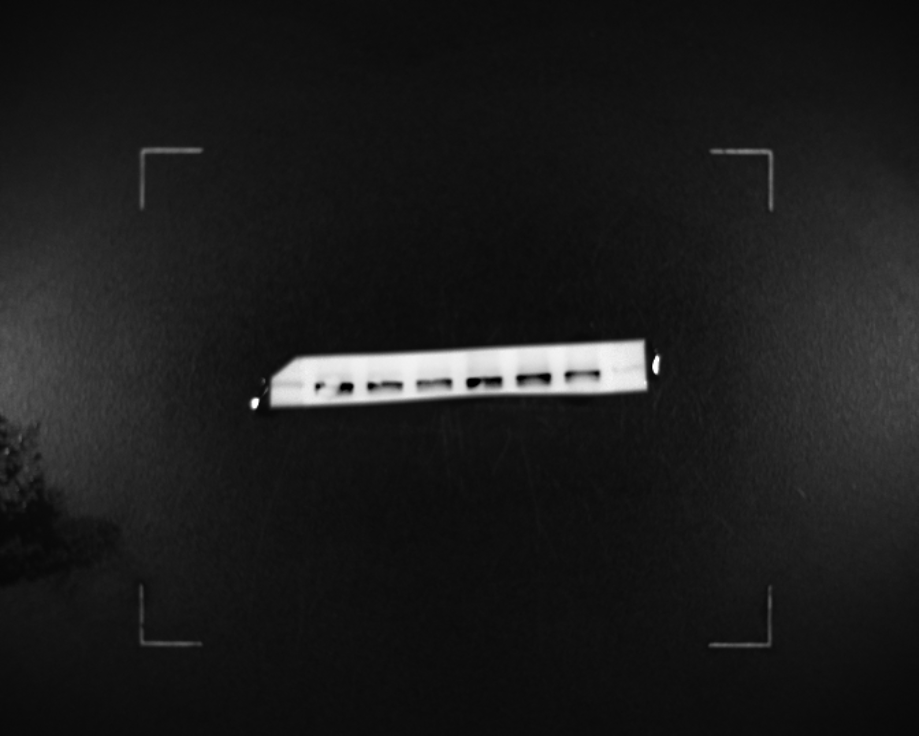


**Figure 3**. The entire original gel of α-SMA-manuscript in Figure 3D.


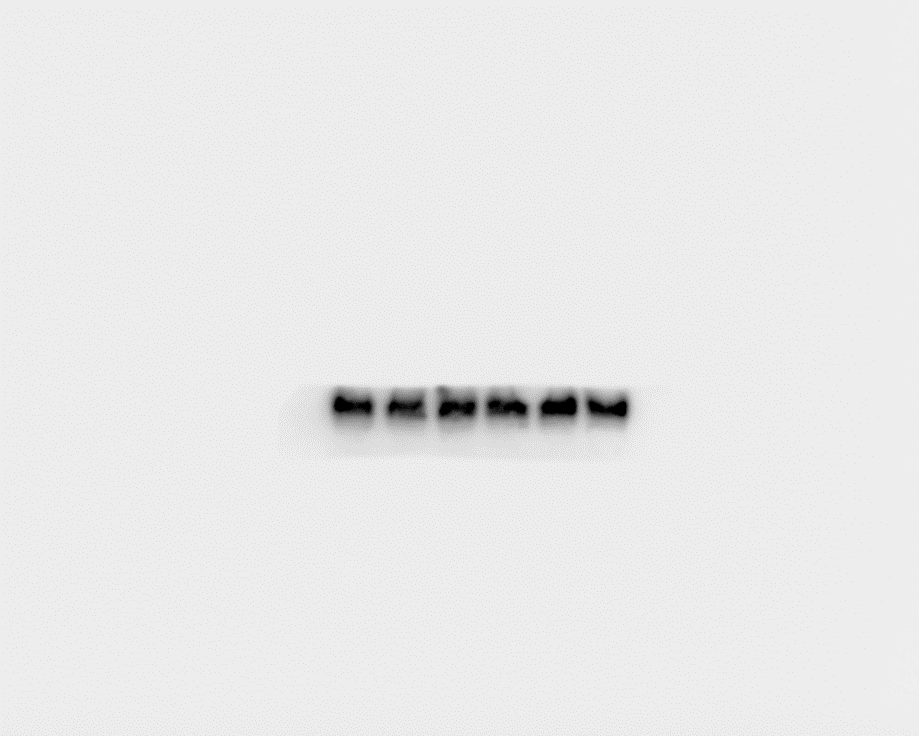


**Figure 3**. The entire original gel of GAPDH-manuscript in Figure 3D.


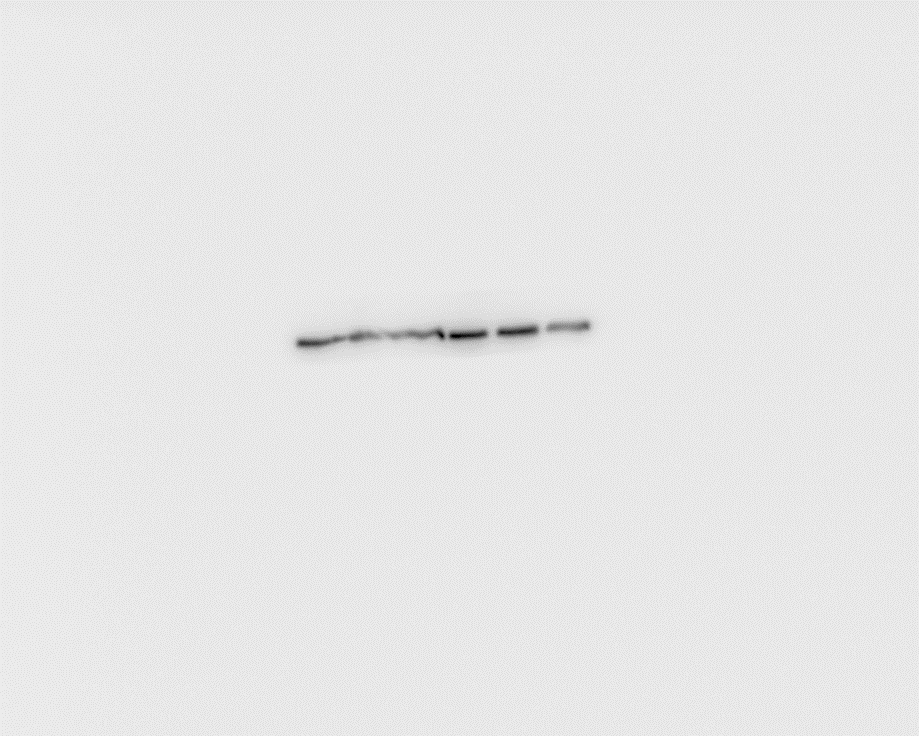


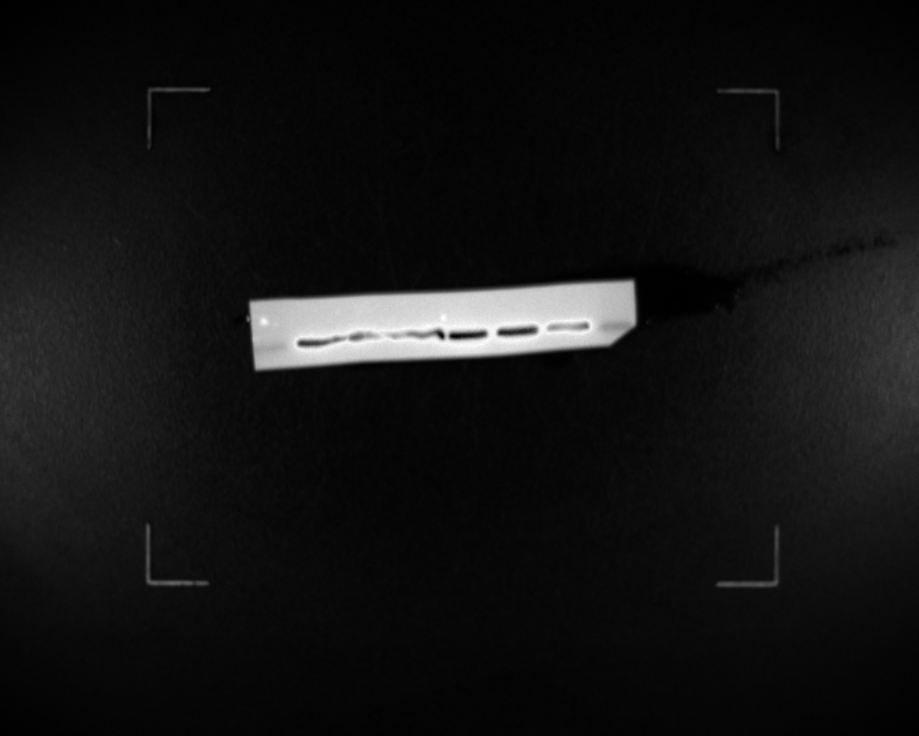


**Figure 3**. The entire original gel of P-smad 2-manuscript in Figure 3G.


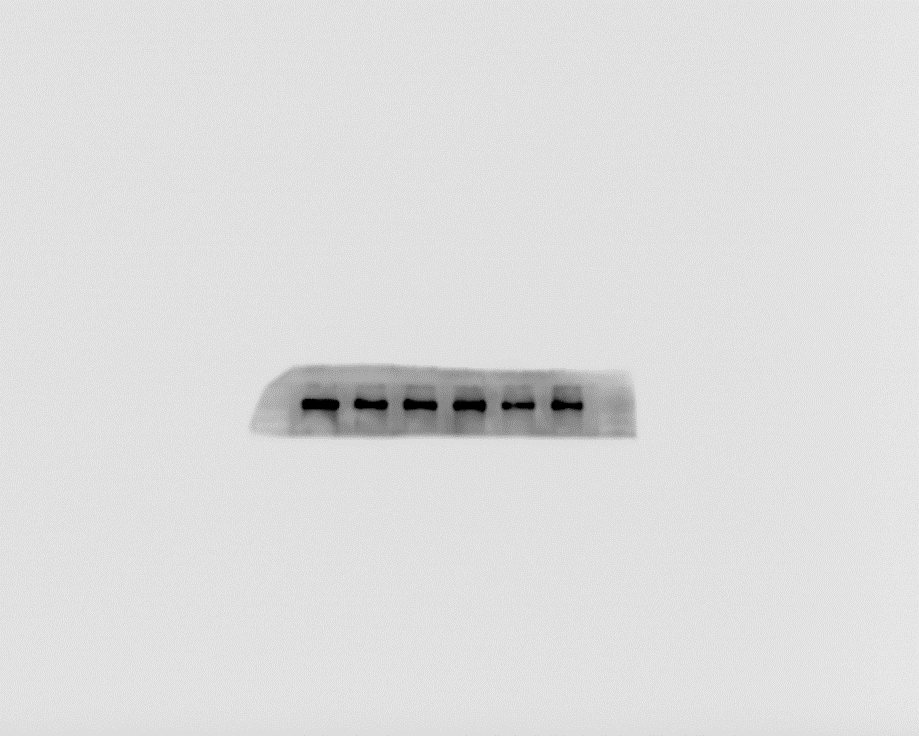


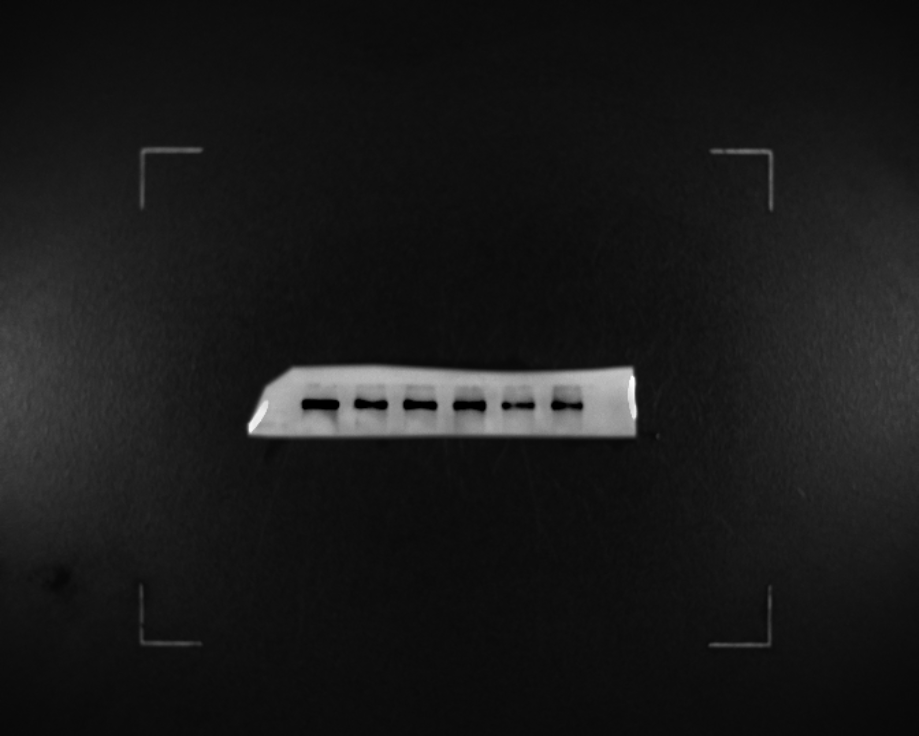


**Figure 3**. The entire original gel of smad 2-manuscript in Figure 3G.


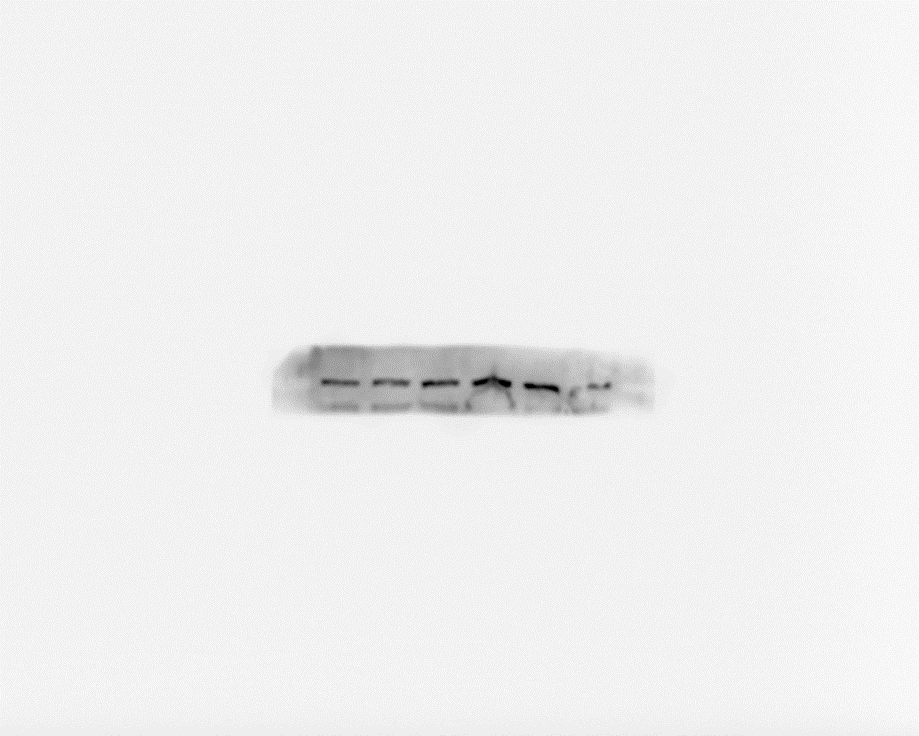


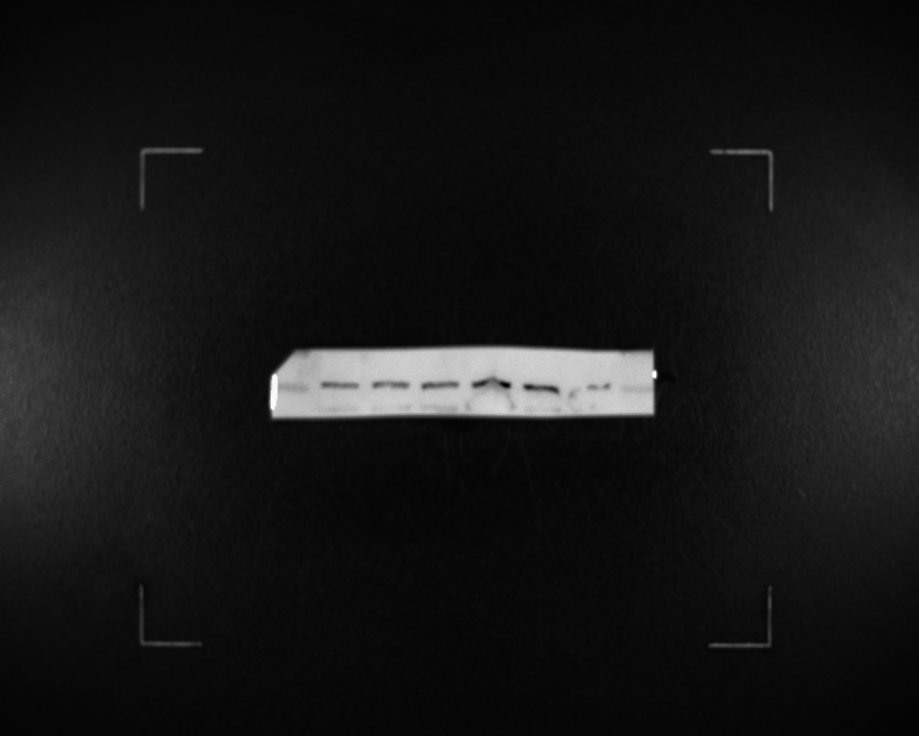


**Figure 3**. The entire original gel of P-smad 3-manuscript in Figure 3G.


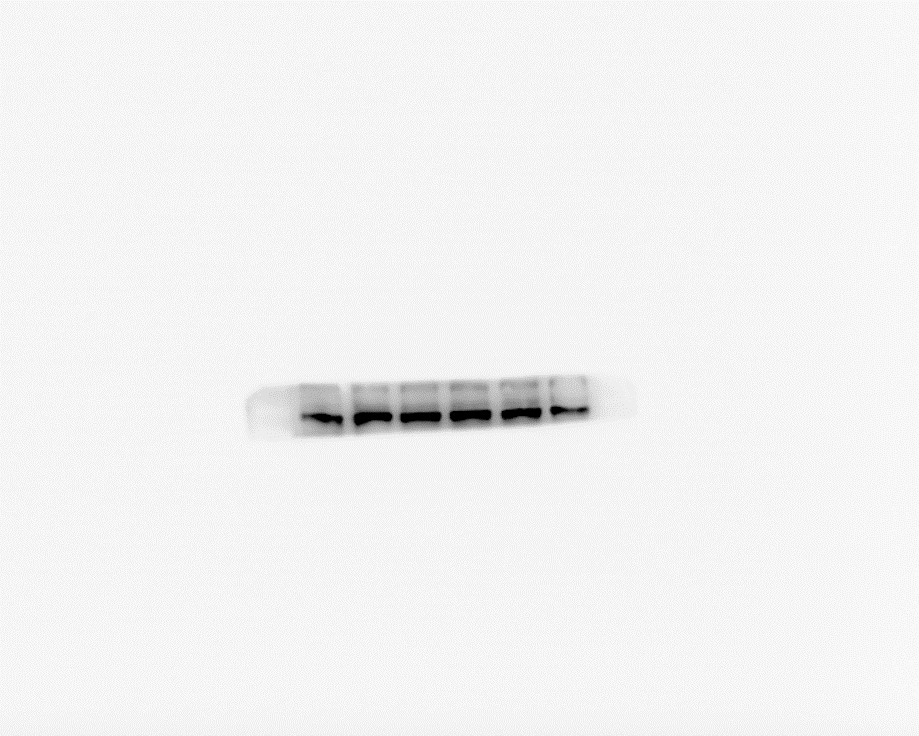


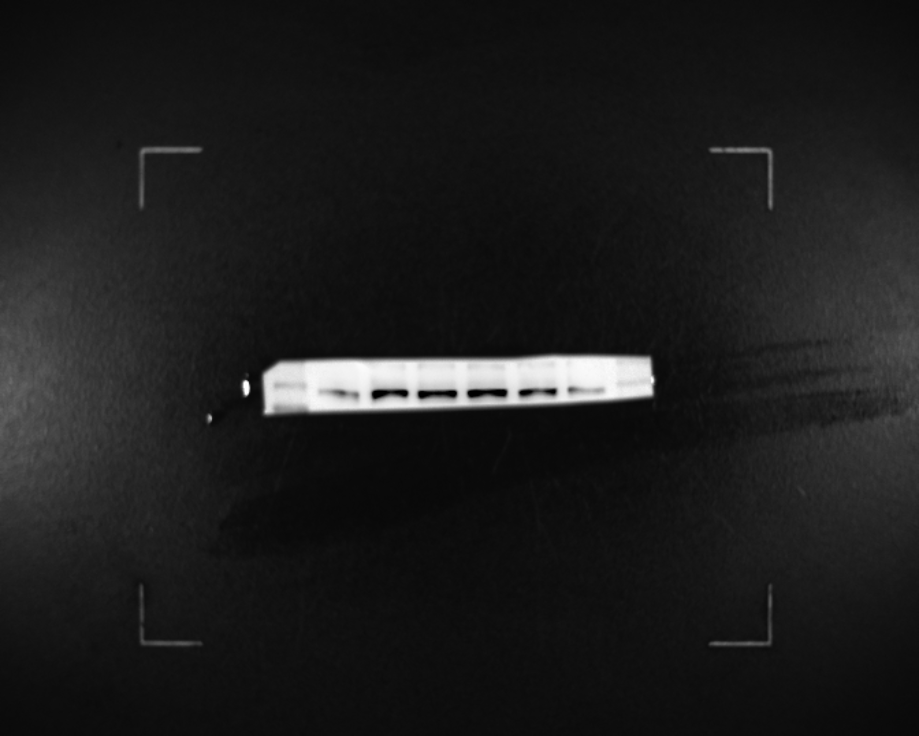


**Figure 3**. The entire original gel of Smad 3-manuscript in Figure 3G.


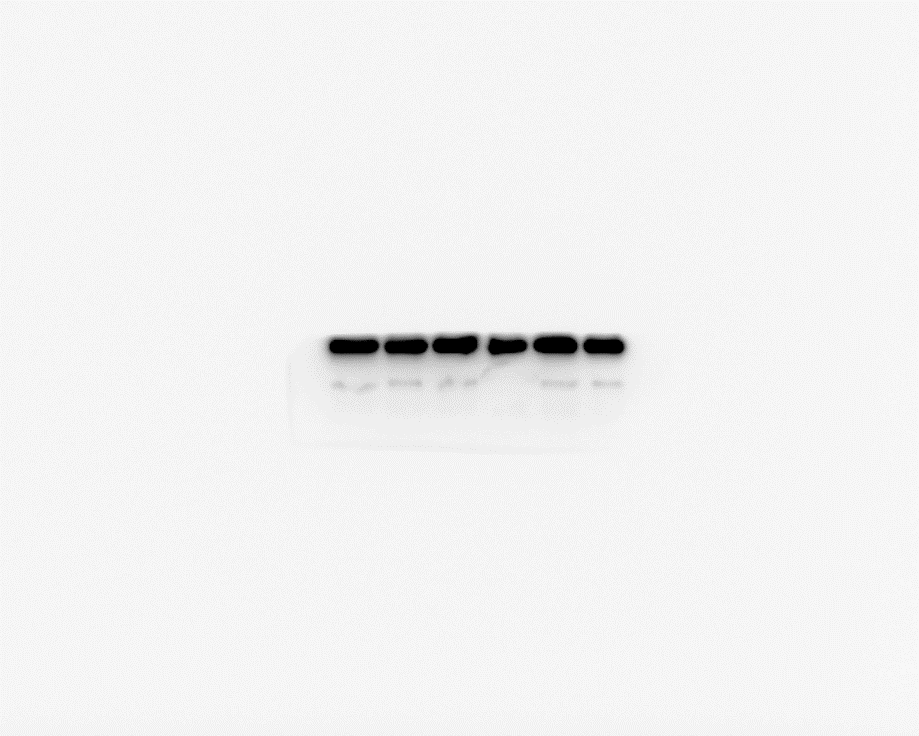


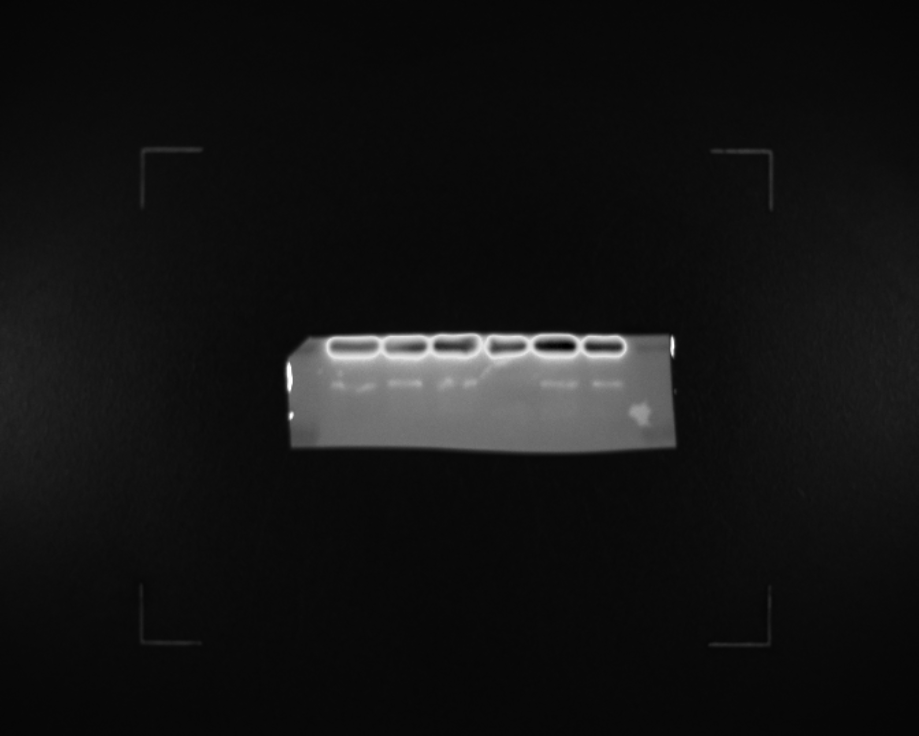


**Figure 3**. The entire original gel of GAPDH-manuscript in Figure 3G.


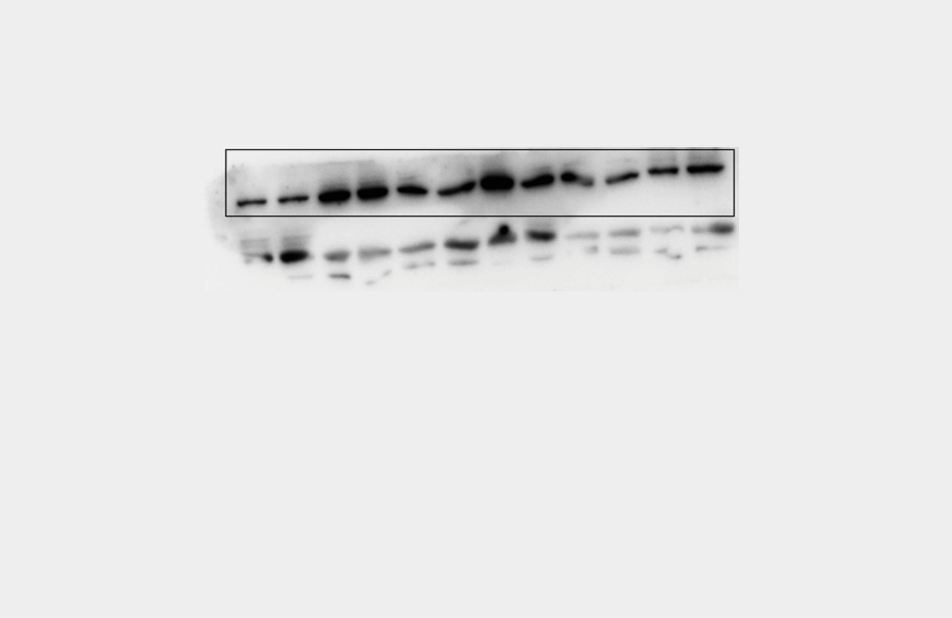


**Figure 4**. The entire original gel of Col1-manuscript in Figure 4D.


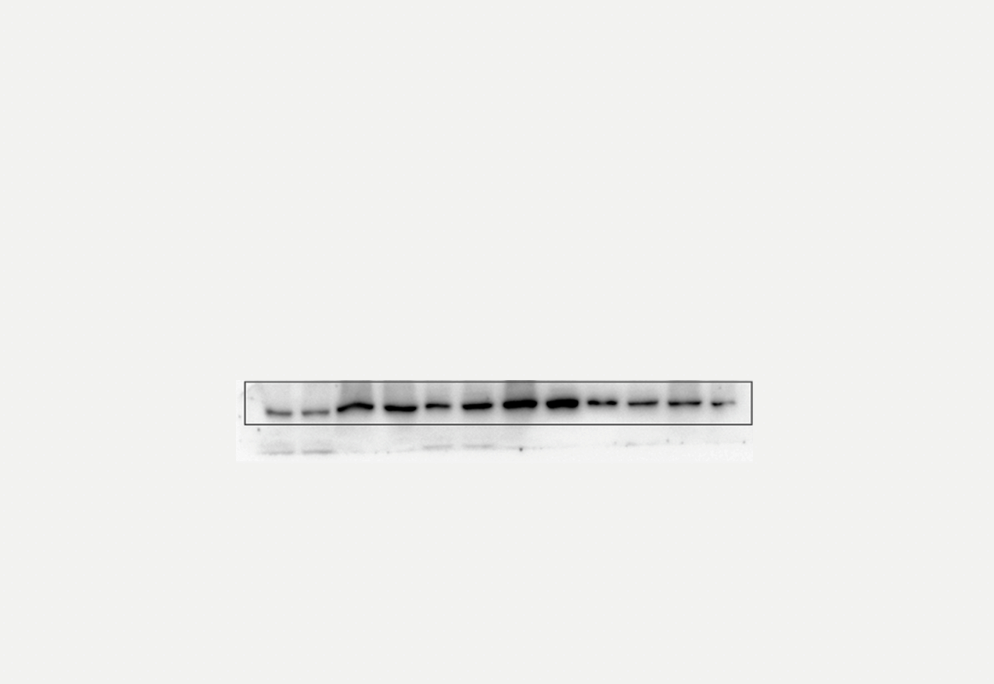


**Figure 4**. The entire original gel of α-SMA -manuscript in Figure 4D.


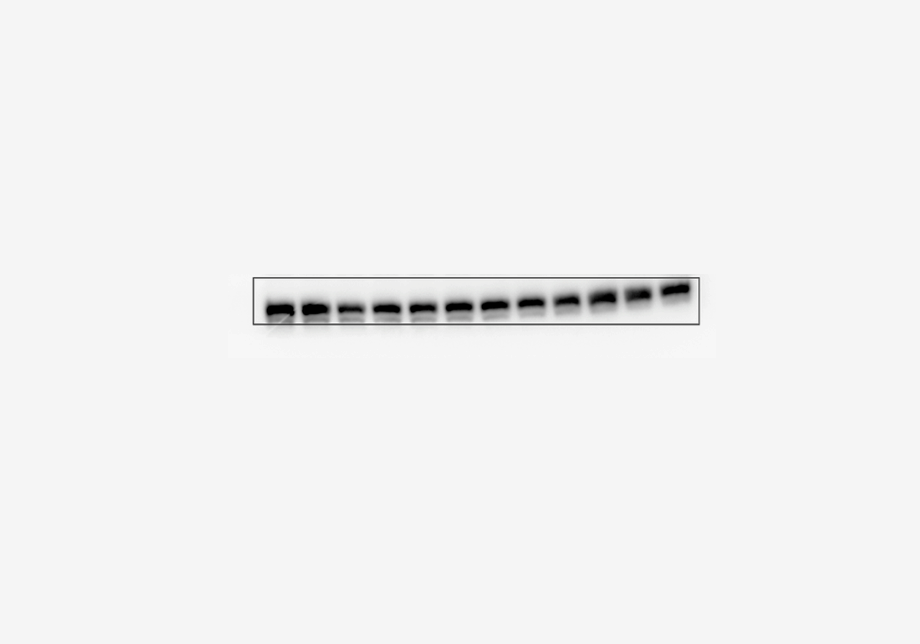


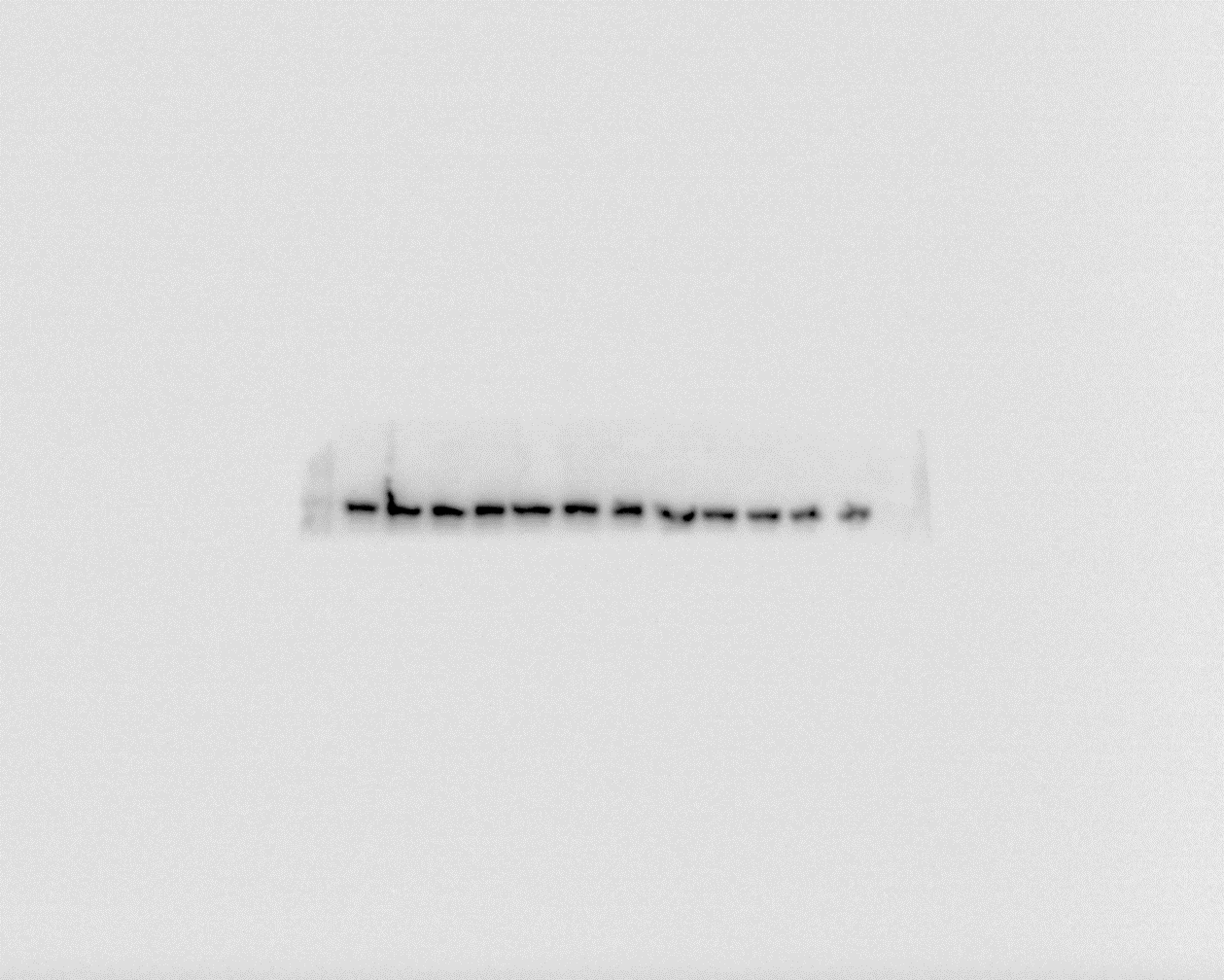


**Figure 4**. The entire original gel of GAPDH -manuscript in Figure 4D.


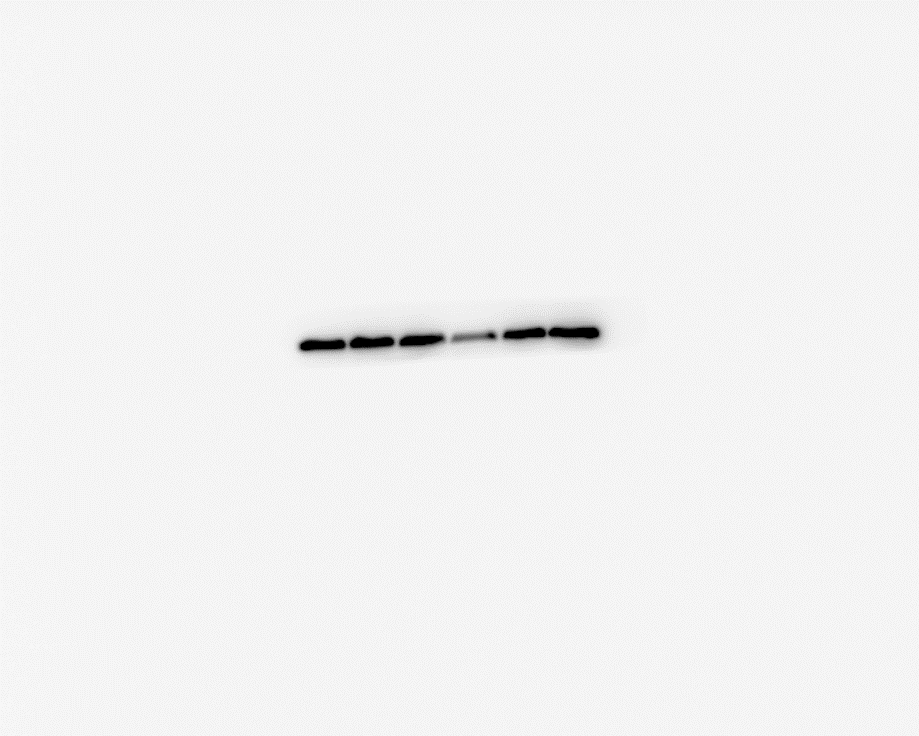


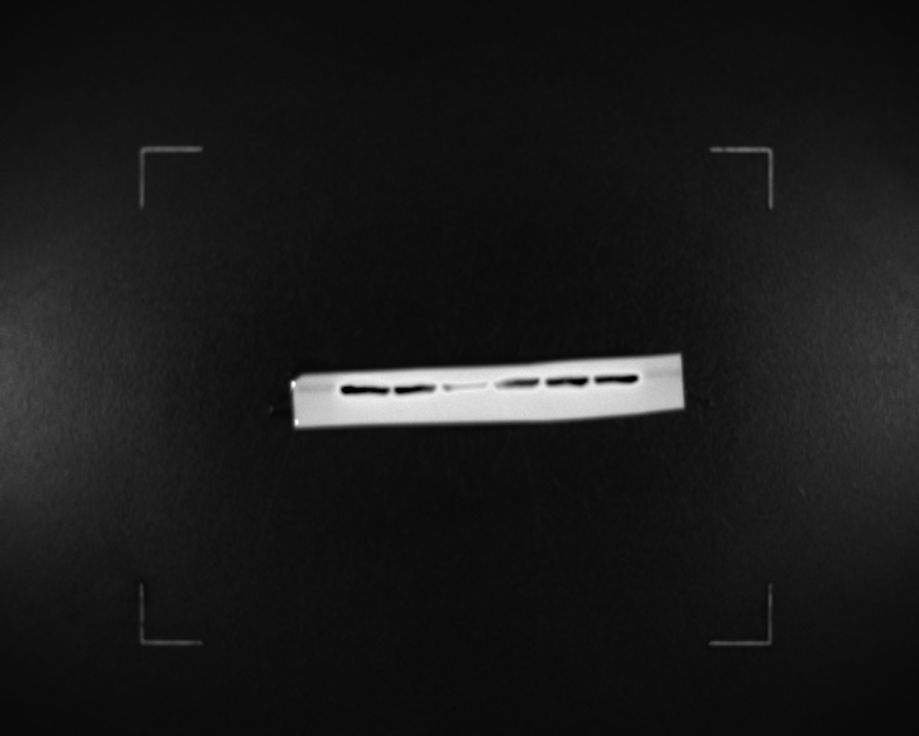


**Figure 5**. The entire original gel of E-cadherin-manuscript in Figure 5D.


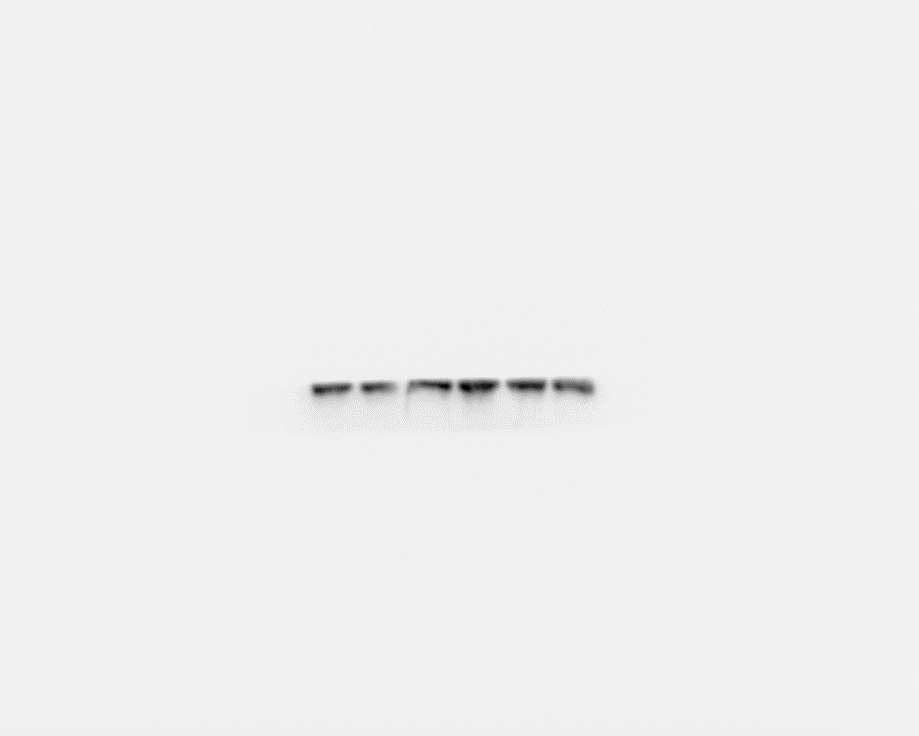


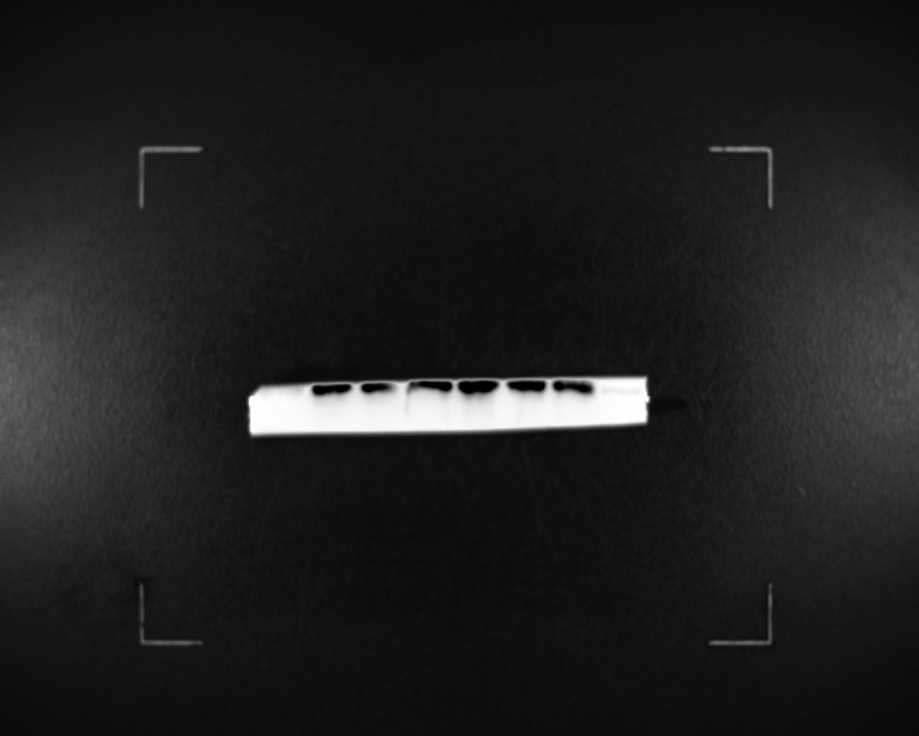


**Figure 5**. The entire original gel of Vimentin-manuscript in Figure 5D.


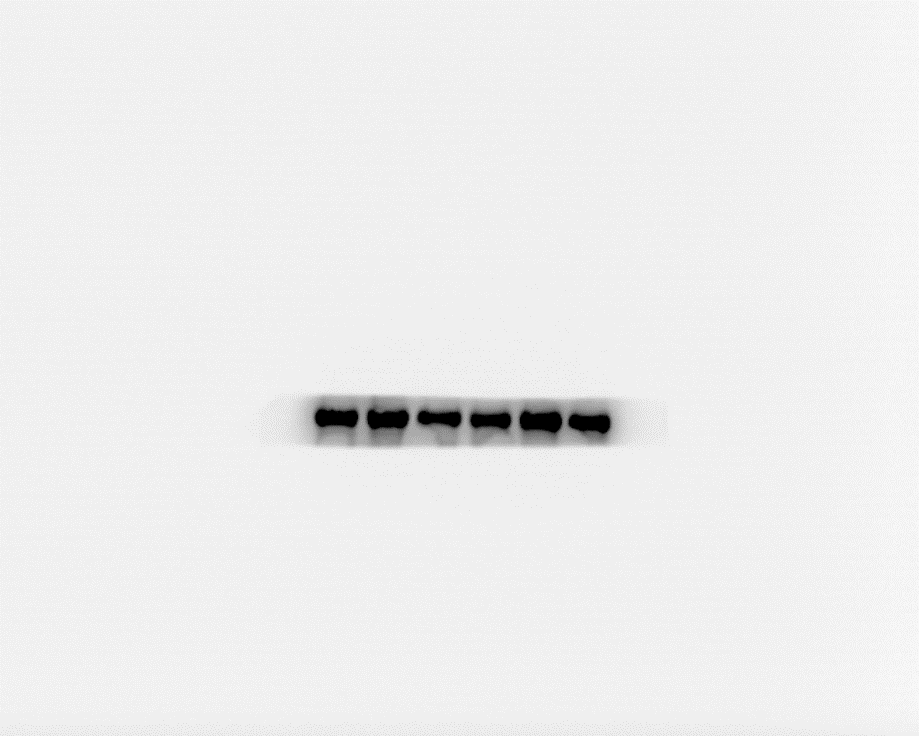


**Figure 5**. The entire original gel of GAPDH-manuscript in Figure 5D.


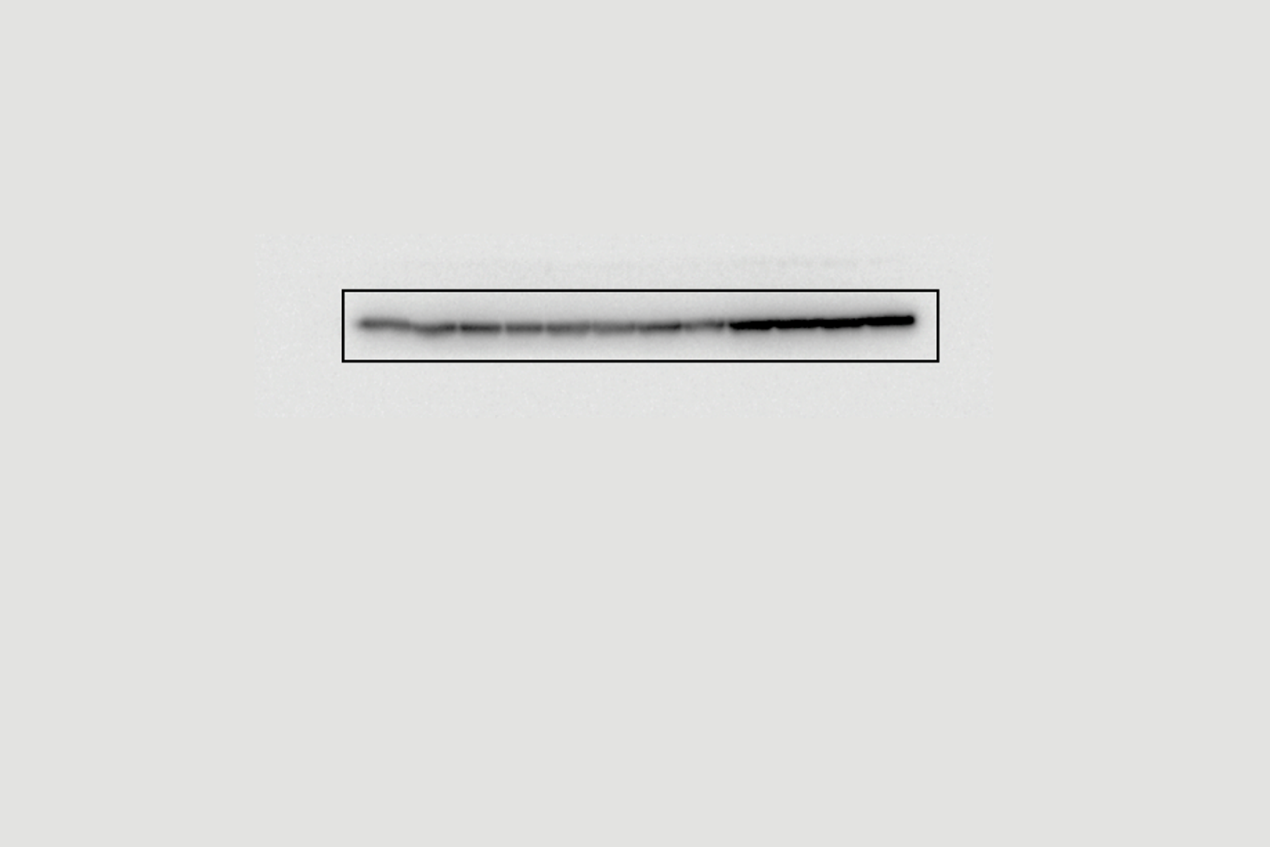


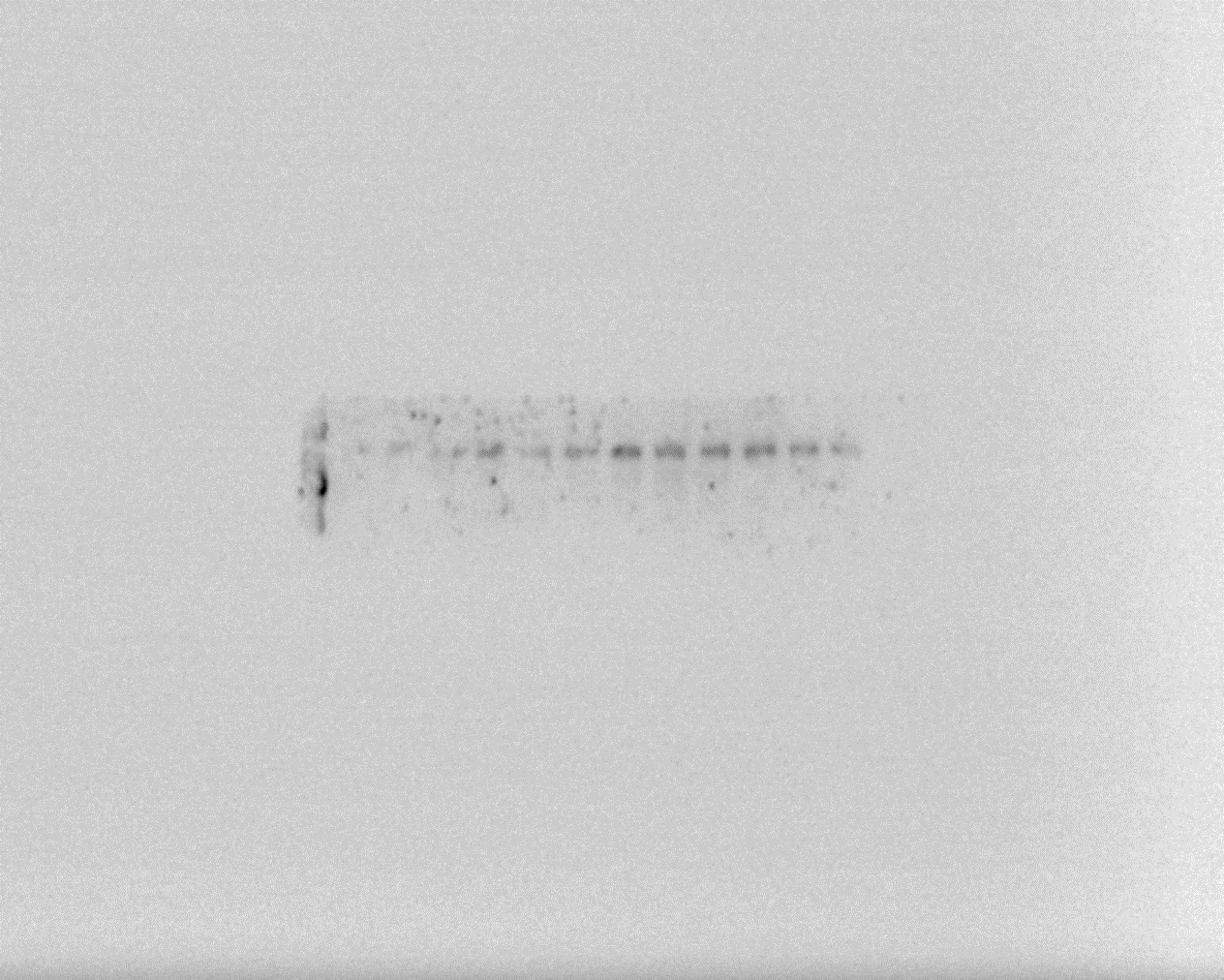


**Figure 6**. The entire original gel of E-cadherin-manuscript in Figure 6D.


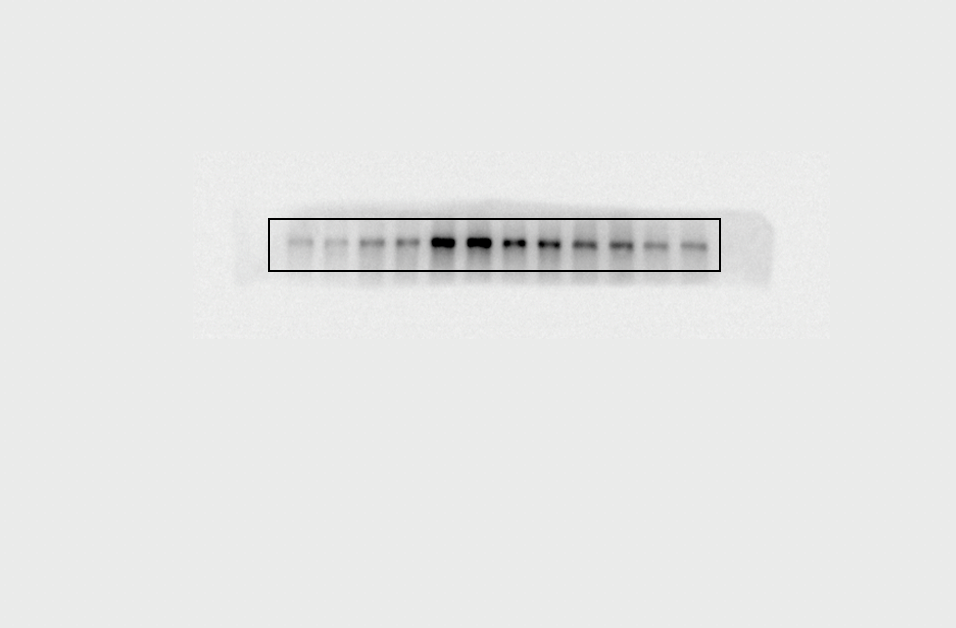


**Figure 6**. The entire original gel of Vimentin-manuscript in Figure 6D.


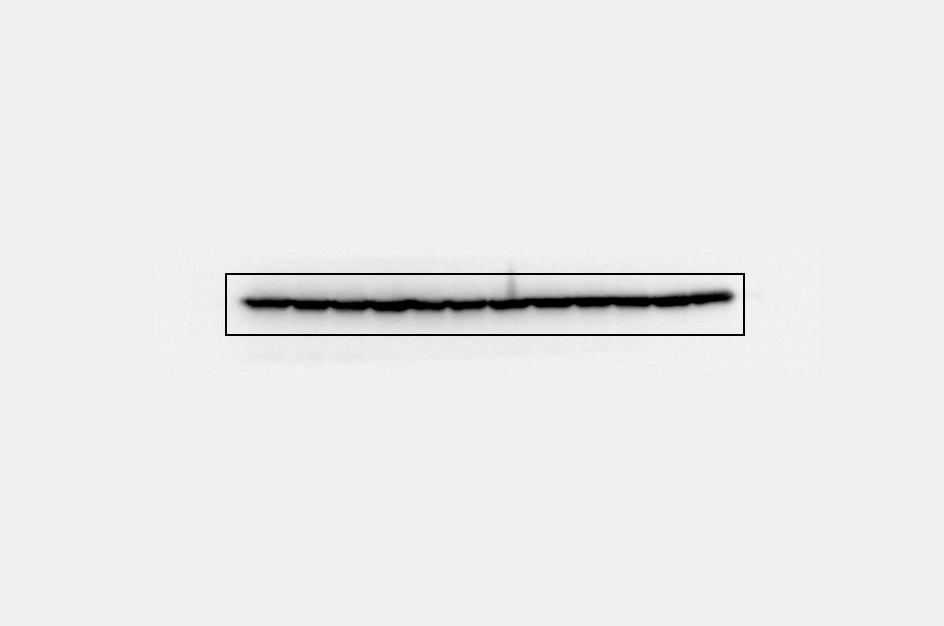


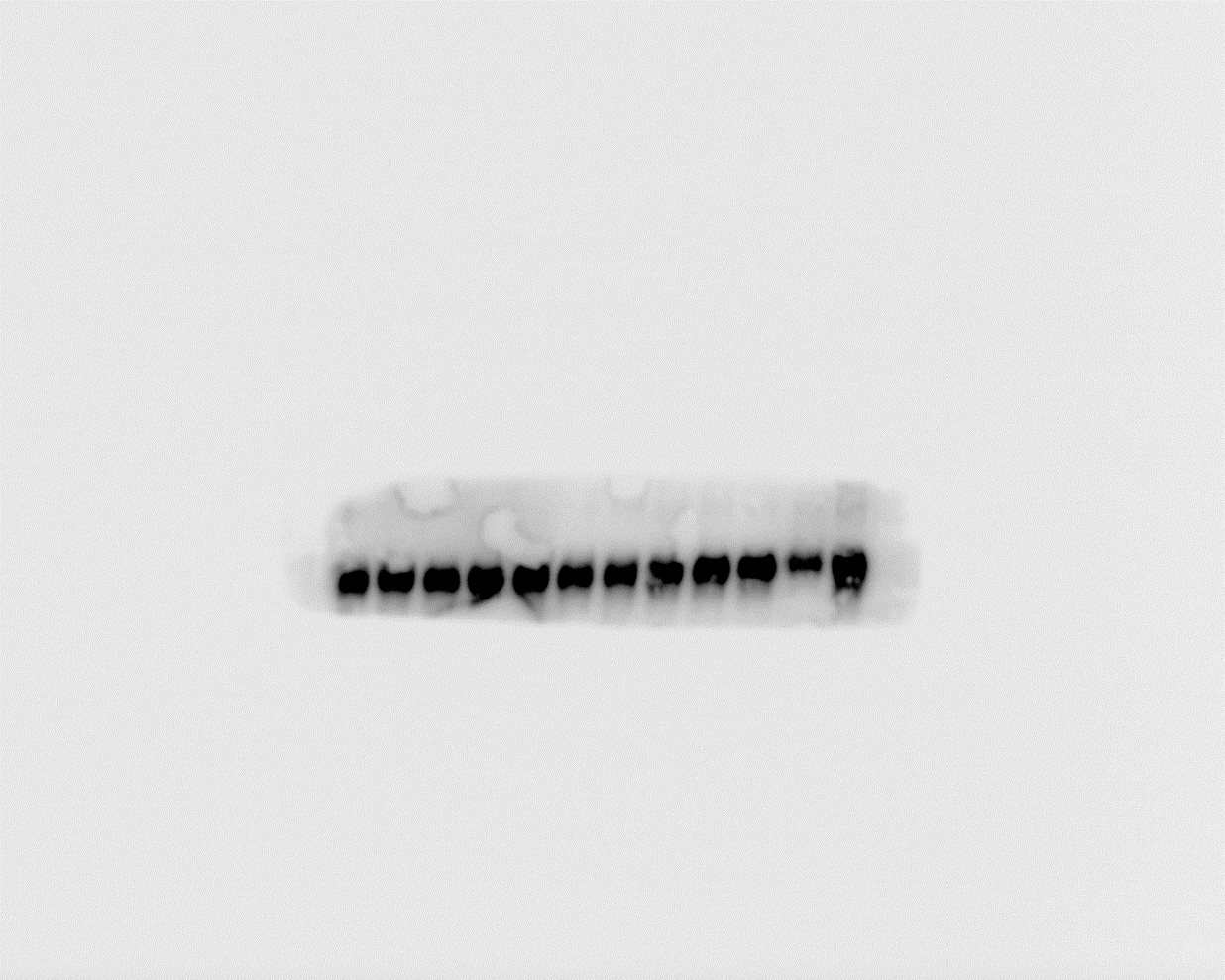


**Figure 6**. The entire original gel of GAPDH-manuscript in Figure 6D.
